# Supplementary material for: A new normalization for Nanostring nCounter gene expression data
Source: Nucleic Acids Res. 2019 May 22;47(12):6073–83. doi: 10.1093/nar/gkz433 (PMC6614807; doi:10.1093/nar/gkz433)
Supplement: gkz433_Supplemental_Files [file gkz433_supplemental_files.docx]

**Supplementary File**

**A new normalization for Nanostring nCounter gene expression data**

Ramyar Molania^1,2,3^, Johann A. Gagnon-Bartsch^4^, Alexander Dobrovic^1,5,6,7^, Terence P Speed^2,8*^.

^1^Translational Genomics and Epigenomics Laboratory, Olivia Newton-John Cancer Research Institute, Heidelberg, Victoria, 3084, Australia;

^2^Bioinformatics Division, Walter and Eliza Hall Institute of Medical Research, Parkville, Victoria, 3052, Australia;

^3^Department of Medicine, University of Melbourne, Austin Health, Heidelberg, Victoria, 3084, Australia;

^4^Department of Statistics, University of Michigan, Ann Arbor, Michigan, MI 48109, USA;

^5^School of Cancer Medicine and Molecular Cancer Prevention program, La Trobe University, Bundoora, Victoria, 3086, Australia;

^6^Department of Clinical Pathology, University of Melbourne, Parkville, Victoria, 3010, Australia;

^7^University of Melbourne Department of Surgery, Austin Health, Heidelberg, Victoria, 3084, Australia

^8^Department of Mathematics and Statistics, University of Melbourne, VIC, 3010, Australia.

*Author to whom correspondence should be addressed: **Terence P Speed**,

Tel: +61 3 934452525

Fax: +61 3 9347 0852

Email: terry@wehi.edu.au

# Lung cancer study

Our in-house Nanostring lung cancer data was part of a study of the expression of DNA repair genes in lung adenocarcinoma (LUAD). A customized panel of 587 genes was profiled using RNA extracted from formalin-fixed paraffin embedded lung adenocarcinoma specimens from 96 patients (some patients with more than one core), and from 12 samples of normal lung tissue. As well as using the vendor-supplied NEG and POS spiked-in controls, a panel of 13 housekeeping genes was selected from the literature based on stability and expression levels across the tissue types of interest. A consistent protocol was used for sample processing and RNA extractions, and data was generated using 15 nCounter cartridges (Figure S.1a). The majority of the normal samples were run in one cartridge a year after the rest of the experiment (Figure S.1a). Several different technical replicate samples (17 duplicates and 6 triplicates) amounting to 29 additional assays (18% of a total of 162) were included within and between cartridges (Figure S.1b).


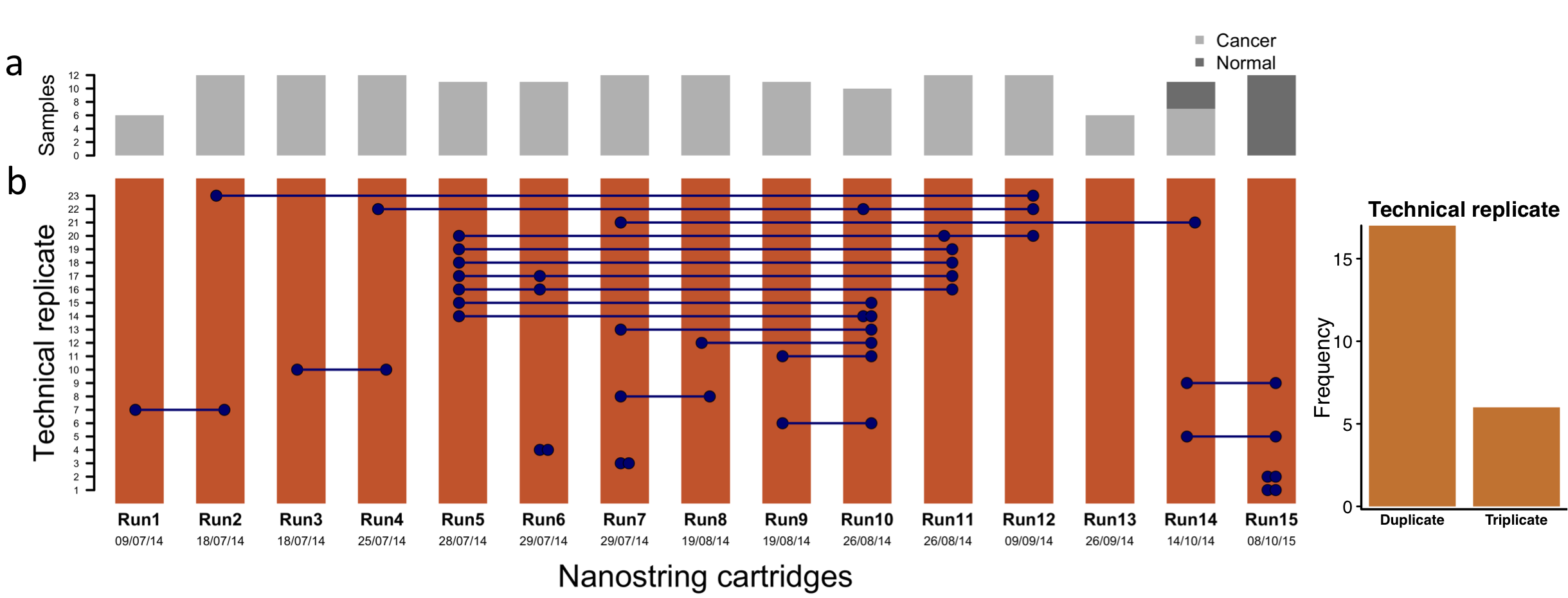


**Figure S1.** Study design of the Nanostring lung cancer study. **a)** The frequency of normal and cancer samples within each nCounter cartridge; most of the cartridges include 12 samples. Normal and cancer samples are not evenly distributed across cartridges. **b)** The distribution of technical replicate samples within and between cartridges. Points connected with a horizontal-solid line represent one technically replicated sample. Technical replicate samples used include 17 duplicates and 6 triplicates.


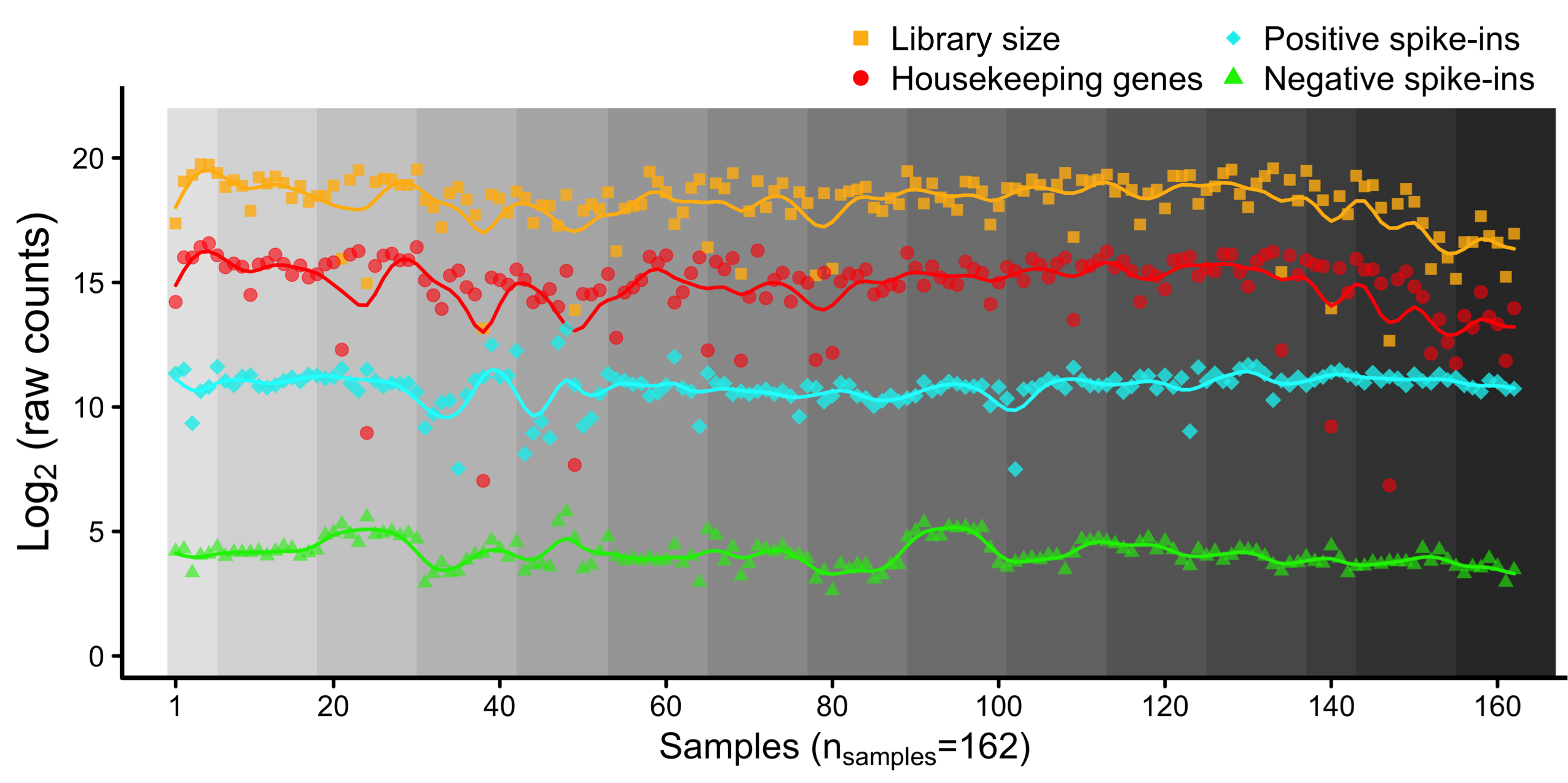


**Figure S2.** Behavior of the spike-in controls in the Nanostring lung cancer study. The average of the negative and positive spike-in log counts, the average log counts of the housekeeping genes and the log library sizes across all samples are presented here. The gradient of gray represents different nCounter cartridges. The behavior of the spike-in controls of some samples is unrelated to the quality of samples. The library sizes of samples in the 15th cartridge are one quarter the average of other samples, whereas the averages of the POS and NEG spiked-in controls are stable across cartridges.

In Figure S3, we summarize the results of normalizing our data with the 84 different options provided by nSolver and the NanoStringNorm R package [1]: 3 ways of using the POS probe counts (N, S and GM) × 4 ways of using the NEG probe counts ( N, M, M2SD, Ma) × 7 ways of using the housekeeping genes (N, S, GM, TS, LCGM, TM, TGM). The meanings of these abbreviations can be found in the figure. For fuller details, we refer to analysis nSolver (version 4) software user manual, which can be found at <https://www.nanostring.com/application/files/6415/1789/7813/MAN-C0019> 08_nolver_4.0_Analysis_Software_User_Manual.pdf.

c
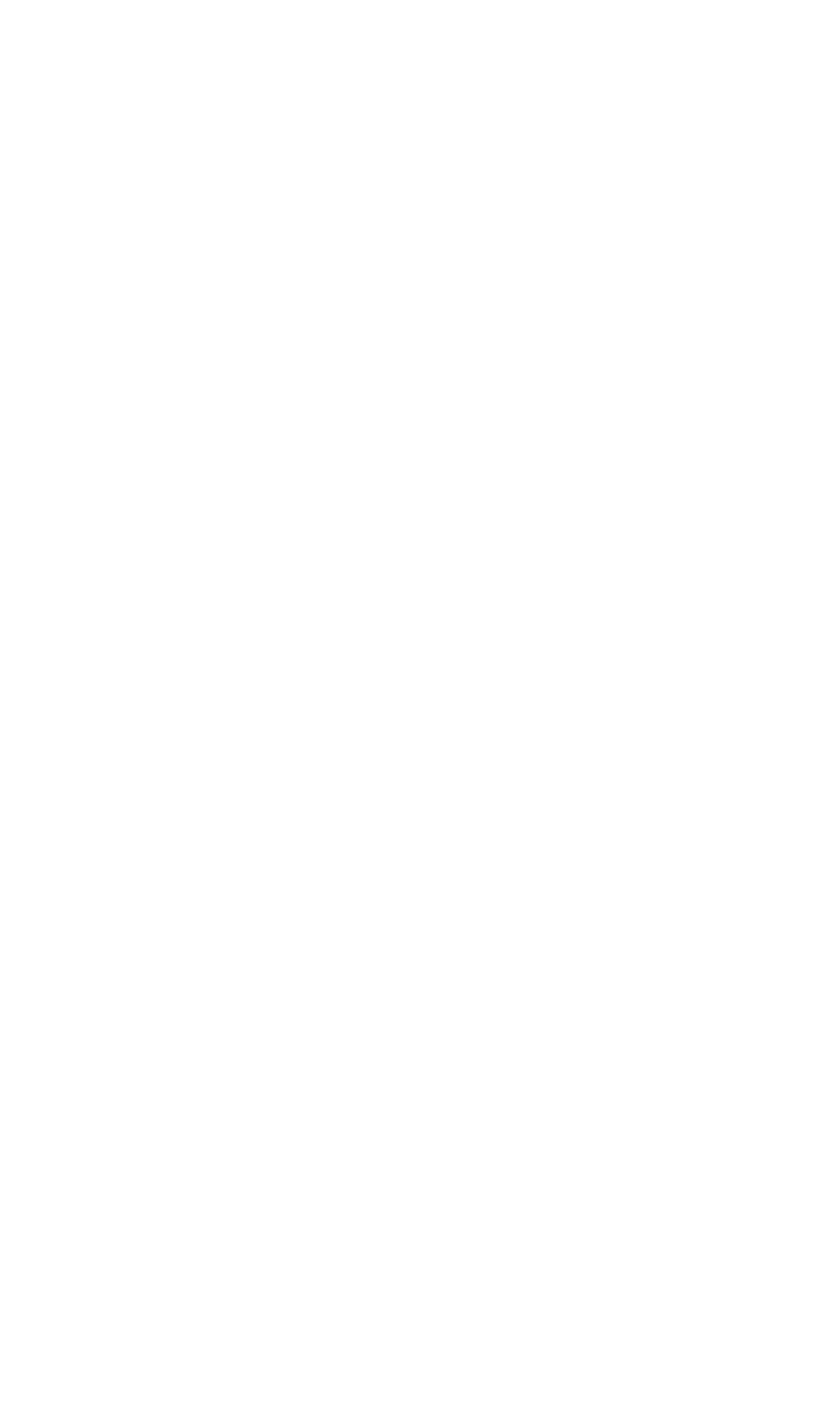


a

b
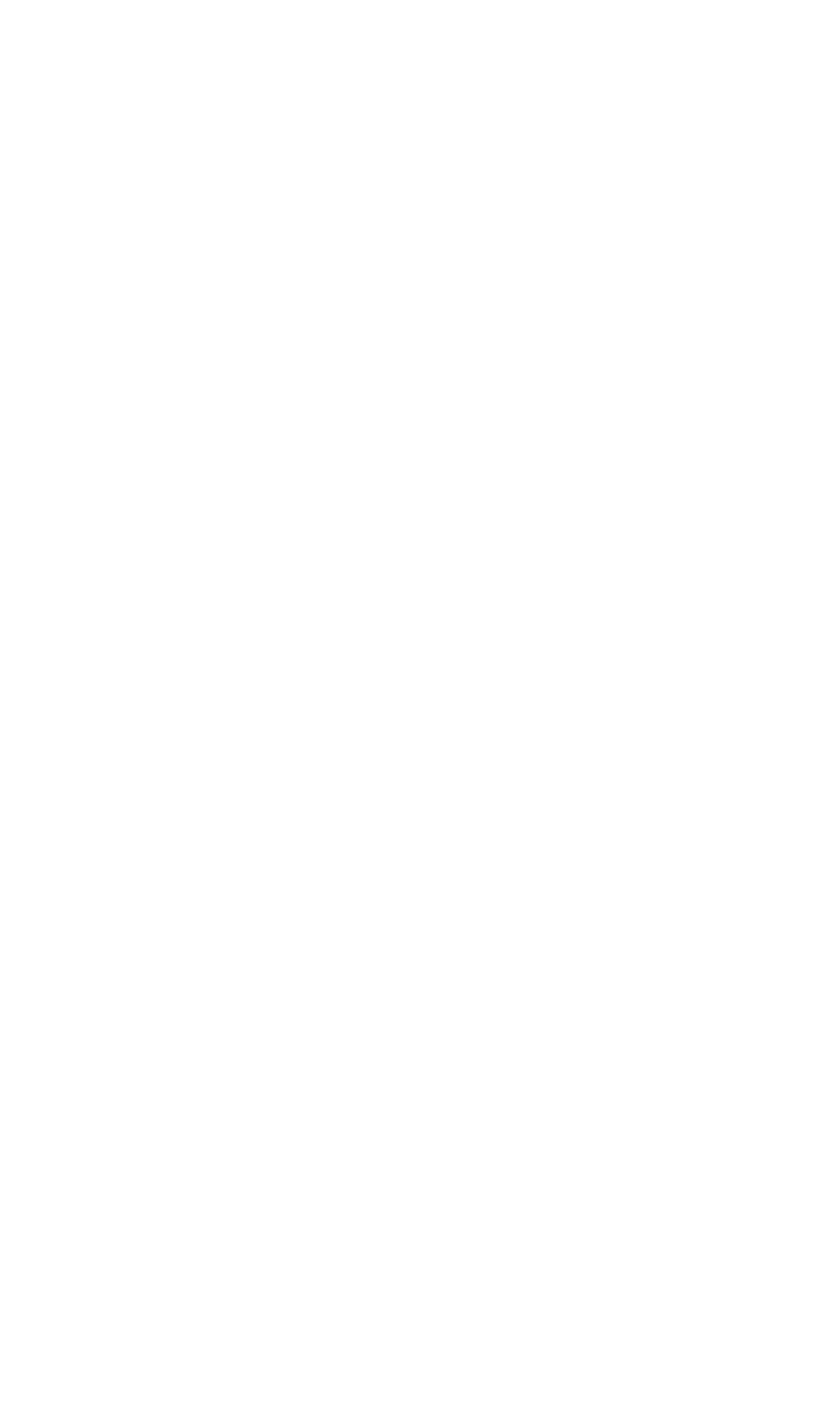


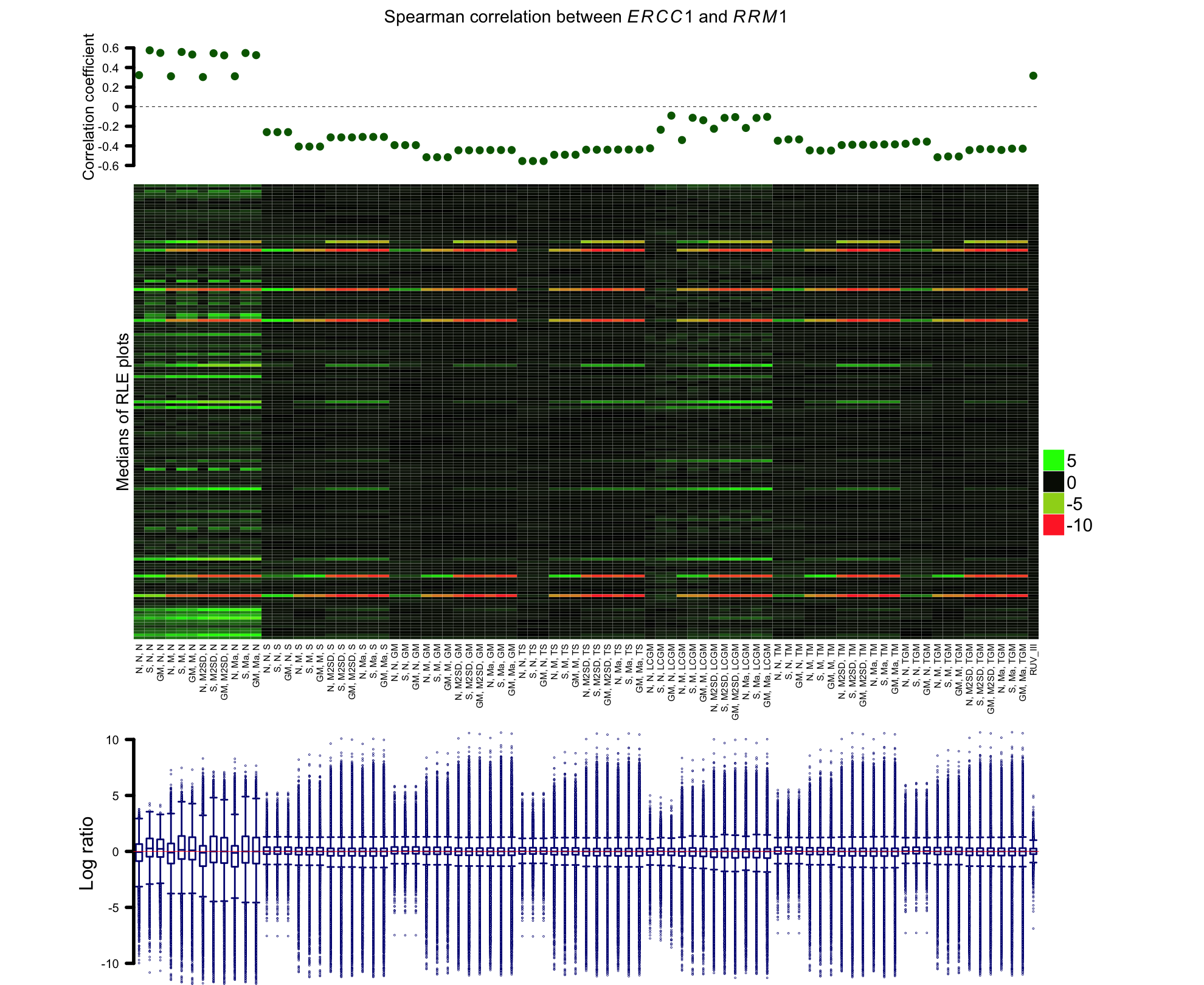


**Figure S3.** Comparing RUV-III with all 84 different normalization options provided by nSolver and the NanoStringNorm R package. **a)** Spearman correlation coefficient between the *ERCC1* and *RRM1* genes. We expect to see a moderate positive correlation between these two genes**. b)** The heatmap shows the RLE plot medians colored by deviation from zero. **c)** Technical replicate agreement (TRA) plots of all technically replicated samples obtained from the different sets of normalized data.

It is apparent from this figure that of these nCounter options, those with no background correction using the NEG measurements give the best results on these measures, that it matters little which of the 3 options are chosen for the use of POS measurements, and that some use of the housekeeping genes is essential. However, not one of the 84 options came close to RUV-III in having centred RLE plots, tight replicate agreement, and a correlation for *RRM1* and *ERCC1* of about r = 0.31.

# Publically available Nanostring datasets­­­

The principal goal of the three Nanostring studies we re-analyse below was the discovery of expression quantitative trait loci (eQTLs), and for doing that, they needed samples from a large number of subjects. However, none of the SNP data for these subjects was publicly available, so that we were unable to link our assessment of their normalizations directly to the determination of eQTLs. As a result, we were forced to give more indirect assessments.

## Inflammatory bowel disease (IBD) study

Peloquin *et al* [2] sought to characterise the expression of IBD-associated risk genes in the setting of active IBD. Terminal ileum (TI) and colonic mucosal tissue were obtained from patients with Crohn’s disease (CD) or ulcerative colitis (UC) and from healthy controls (HC). The goal of the project was to characterize the differences in expression of IBD-associated disease genes in uninflamed and inflamed regions of the TI and colon from CD and UC patients. The majority of the 698 genes studied came from microarray studies of another group, supplemented by 156 genes not on the microarray, chosen for their proximity to tagged risk SNPs and their known expression patterns in cell types of interest in IBD. Analyses included differential expression and variance analysis, weighted gene co-expression network analysis, and eQTL analysis. In our re-examination of their Nanostring data, we focused on differential expression analyses.

In this study, three lots of reagents^[[1]](#footnote-1)^ were used over three years to profile a customized panel of 698 genes (Figure S.4a) in 989 uninflamed or inflamed tissue samples from the colon, rectum or terminal ileum of patients with Crohn’s disease (CD) or ulcerative colitis (UC), and healthy controls. The nCounter assays included the standard 6 POS and 8 NEG spiked-in controls, and a custom set of 15 housekeeping genes. In Figure S.4a we display the distribution of tissue types and disease states across the three batches, and note that it is reasonably balanced, although there are relatively few healthy colon tissue samples in batch 3, and none in batch 4. We excluded 30 samples which they do not have annotations. We found 31 well-distributed replicates (21 duplicates, 5 triplicates and 5 quadruplicate) amounting to 46 additional assays (5% of a total of 959 samples); see Figure S.4b.


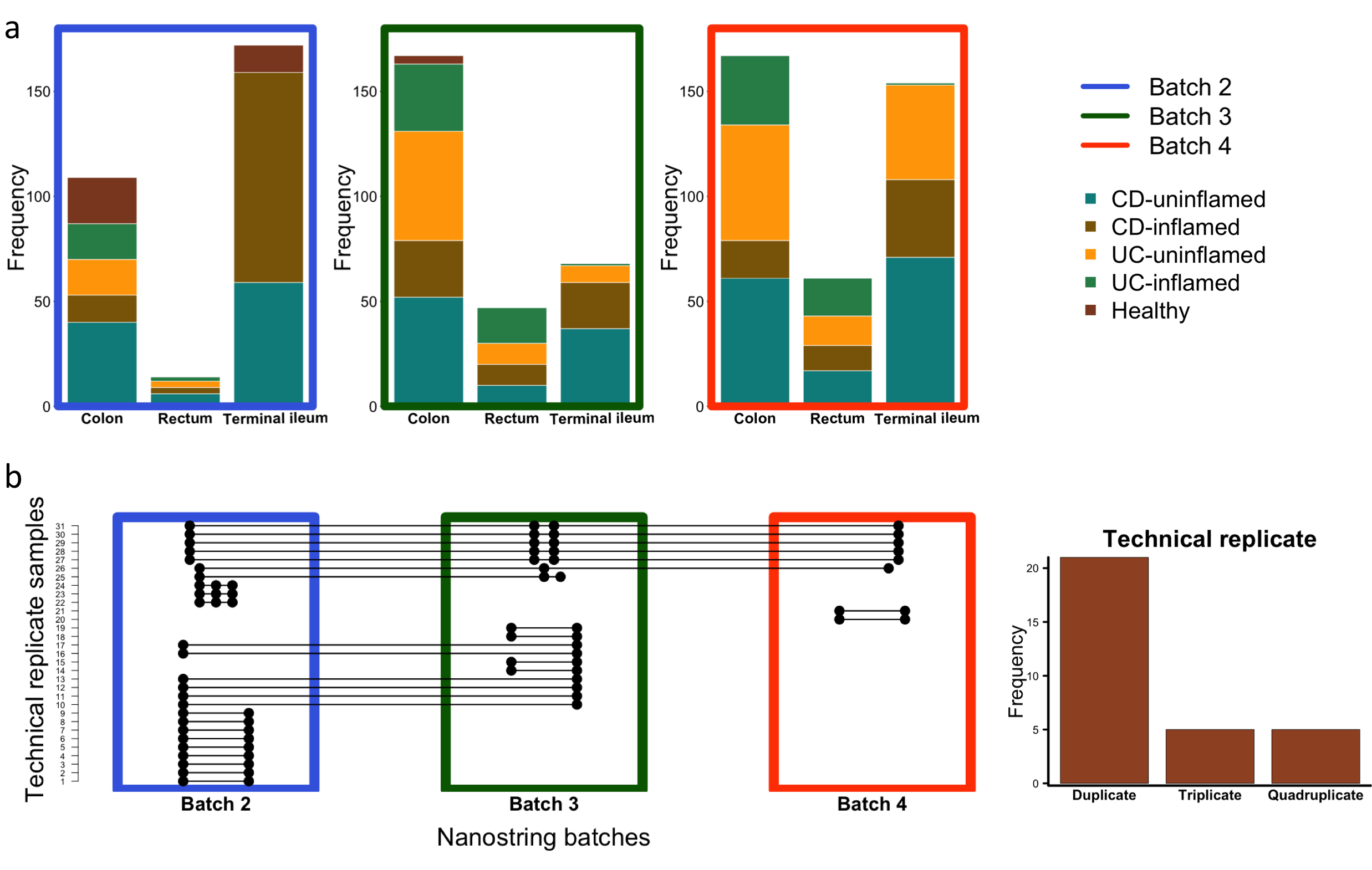


**Figure S4. Study design of IBD study. a)** The distribution of tissues, disease states and healthy individuals across Nanostring batches. Colored boxes represent different batches and stacked bars display frequency of disease states and healthy individuals for each tissue. The majority of healthy colon and all healthy terminal ileum samples are in batch 2. **b)** Allocation of technical replicate samples within and between batches. The technical replicate samples include 21 duplicates, 5 triplicates and 5 quadruplicates, which are reasonably distributed within and between the different batches.

The RLE plots (Figure S.5a) of unnormalized Peloquin data indicate substantial unwanted variation within and between batches. The Peloquin normalization largely removed this variation but the medians of RLE plots are not centered around zero (Figure S.5a) indicating batch effects remain in the Peloquin-normalized data. The RLE plots of RUV-III normalization (Figure S.5a) show that this unwanted variation is effectively removed.

The average plot of the Peloquin data (Figure S.5b) shows the negative and positive spiked-in controls were roughly in agreement with the library sizes and average of housekeeping genes, though occasionally showing considerable variability. The technical replicate agreement plot (Figure S.5c) shows that the RUV-III normalization leads to better agreement between technical replicate samples when compared with that of the Peloquin-normalized data.


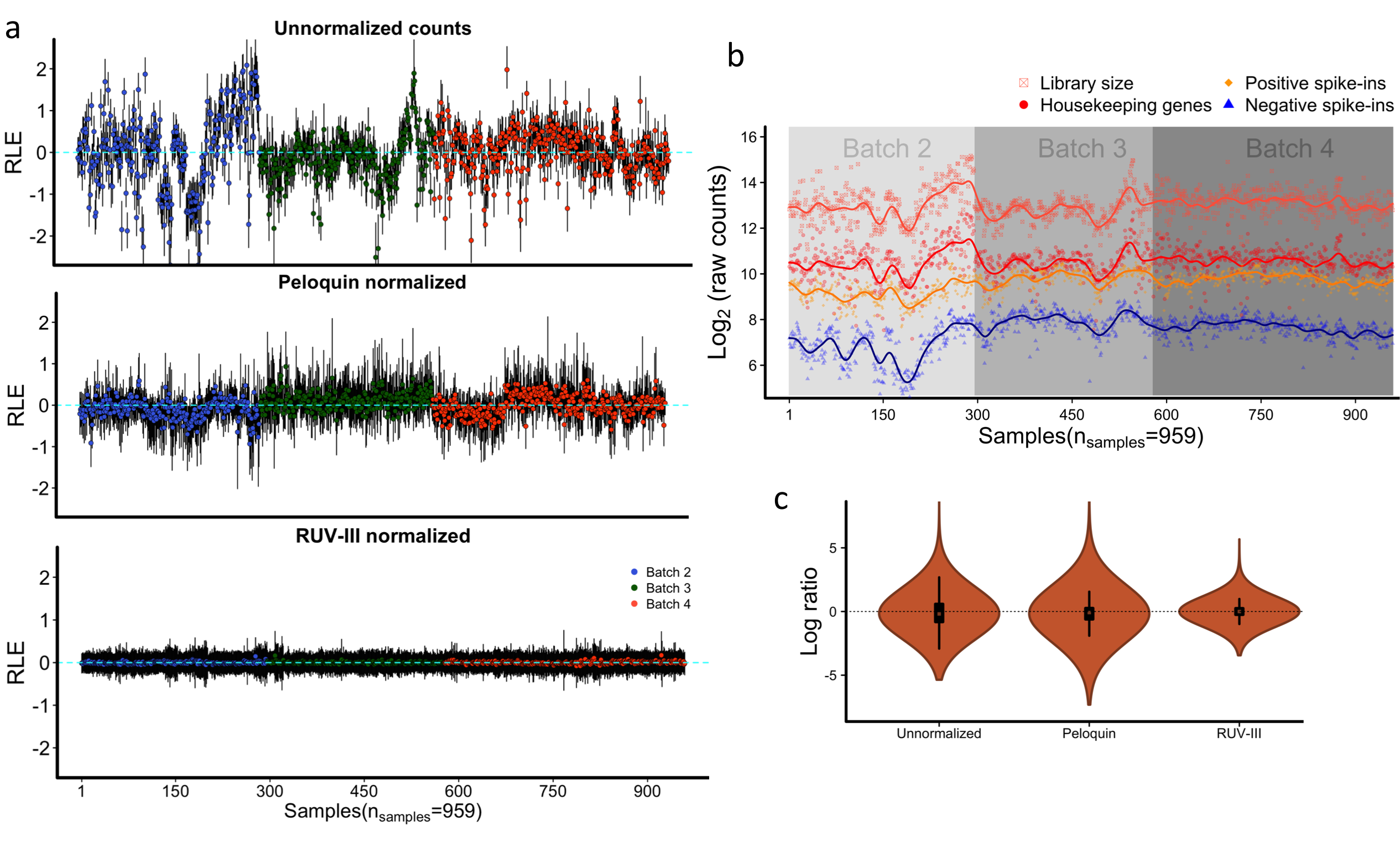


**Figure S5. The performance of different normalization methods on Peloquin *et al* Nanostring data. a)** The RLE plots of unnormalized data, Peloquin-normalized and RUV-III normalized data. The interquartile range and medians of RLE boxplots are displayed. Samples are ordered chronologically based on running the Nanostring cartridges. **b)** The average plot of the Peloquin *et al* Nanostring data. c) The technical replicate agreement (TRA) plots obtained from unnormalized, Peloquin-normalized and RUV-III normalized data. Box plots and kernel density plots are presented for all TRA plots.


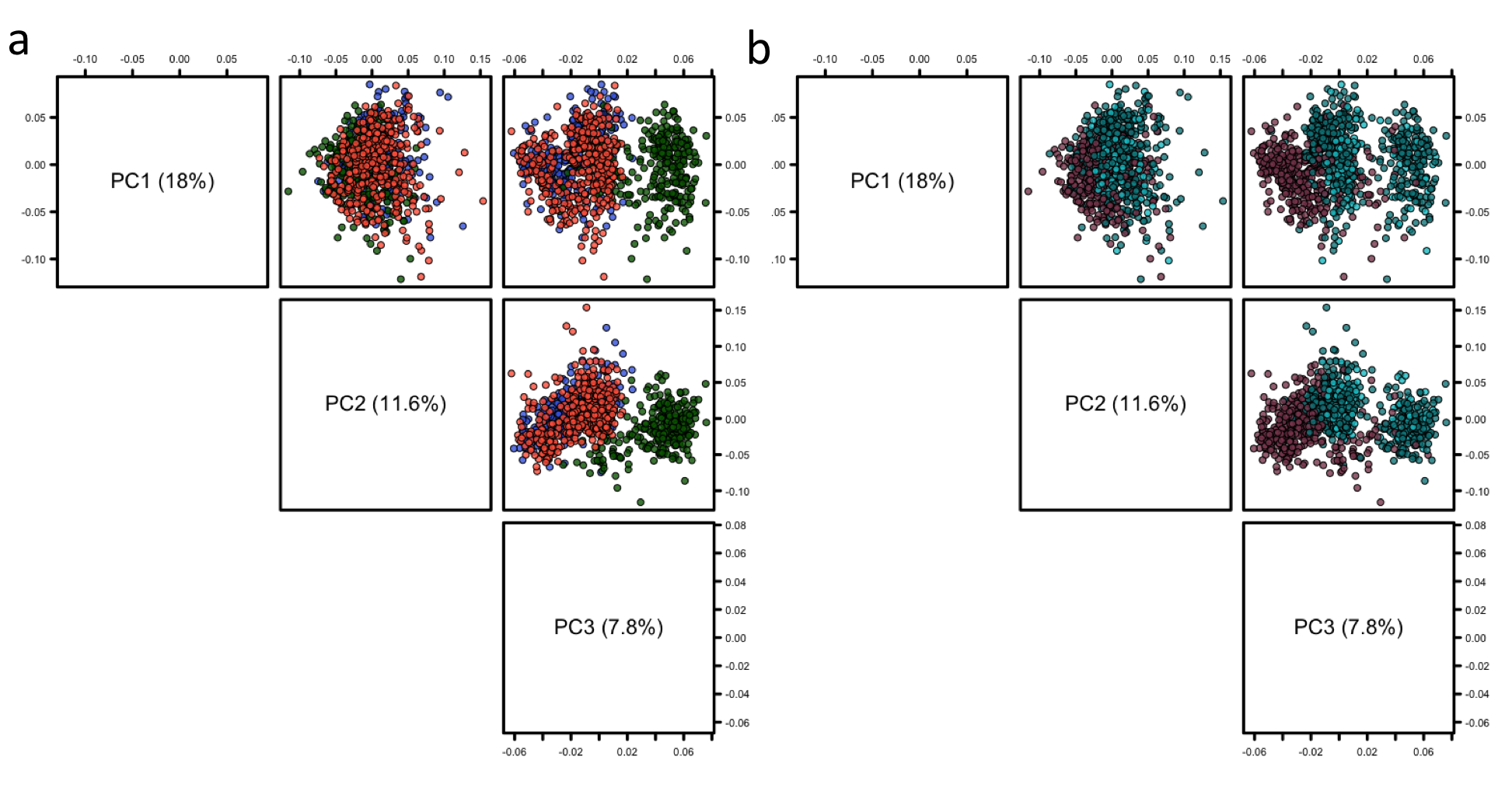


**Figure S6. PCA plots with the correlation matrix of Peloquin normalized Nanostring data. a)** The first three PCs of the correlation matrix of Peloquin normalized Nanostring data colored by reagent lots (left) and by different tissues (right).

We performed differential expression (DE) analysis of each unique disease state between the different Nanostring batches to illustrate the effects of unwanted variation (Figure S.7). Ideally genes should not be differentially expressed between pairs of Nanostring batches. The DE analysis of Peloquin-normalized data showed several significant differentially expressed genes, whereas little evidence of differentially expressed genes was seen in the RUV-III normalized data.


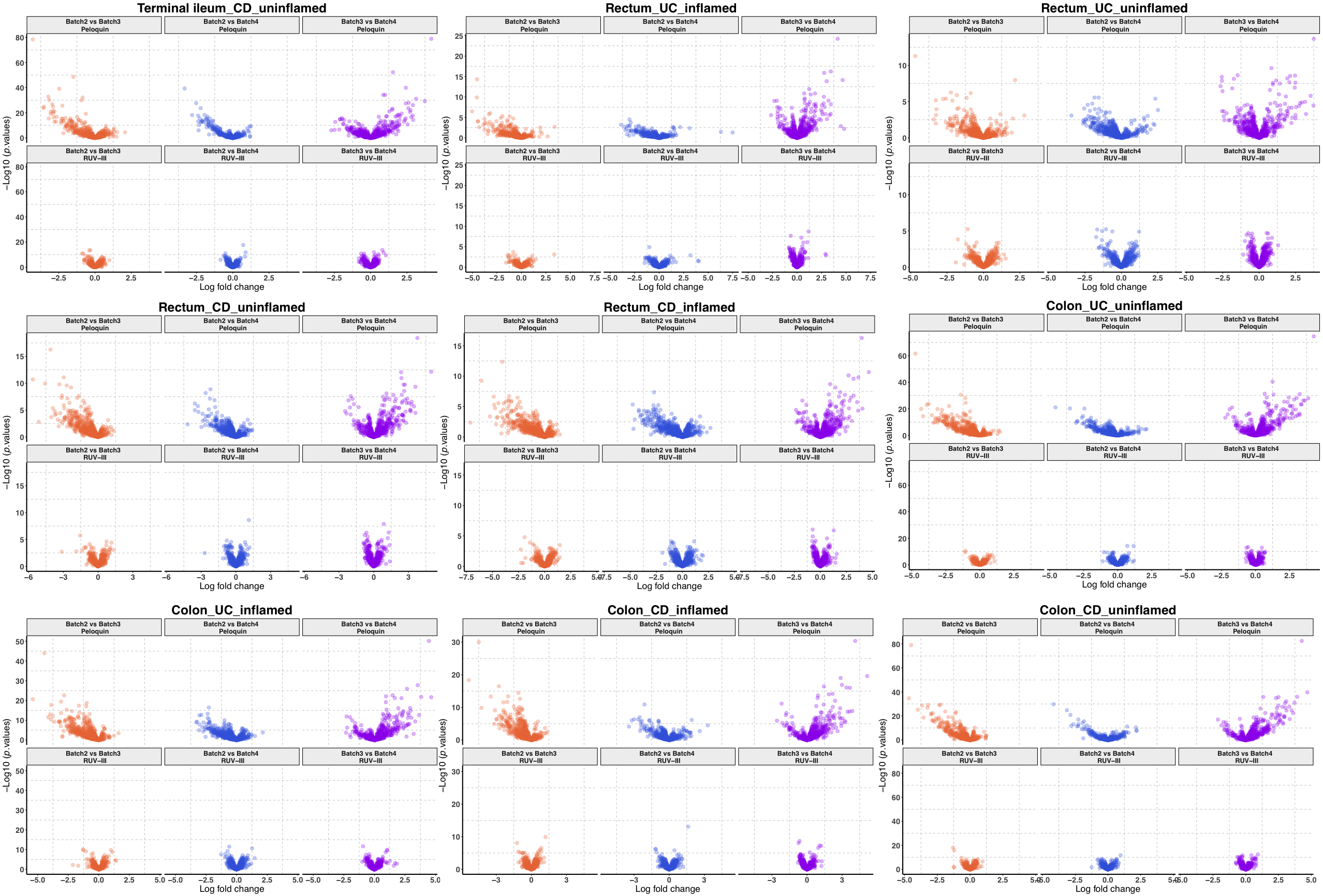


**Figure S7. Volcano plots of DE analysis of 9 unique sample types between pairs of batches.** Differential expression analysis of each unique sample types between pairs of batches were performed on Peloquin-normalized data and RUV-III normalized data. The x-axis represent log fold change and the y-axis are –log_10_ of the unadjusted *p*-values.

**Figure S8. Genes apparently affected by batch effects in the IBD study.** Dot plots of six genes using the Peloquin normalized data. The data seem to be influenced by batch effects in different ways, while the RUV-III normalization largely removes this variation.

## T cell study

Ye *et al* [3] studied CD4^+^ T cells under various conditions in a study aimed at dissecting the contributions of genetics and environment to variation in healthy human T-cell response. Before moving to the Nanostring platform, the authors conducted genome-wide mRNA profiling of suitable T cells using Affymetrix Human Gene 1.0 ST microarrays. They began with a time course study on pooled samples from 6 healthy donors of different genders and ancestries, to select the time points to sample more fully. Next, cells from each of 15 donors were profiled at each of the 5 time points they selected (hereafter called conditions). A panel of 236 genes was chosen on the basis on the second set of microarray data, and these were used in a Nanostring study to assess their variation across 348 healthy subjects.

The Nanostring dataset for this study, which we obtained from GEO, consisted of 1,808 assays generated over 4 months in 2012 and 2013 (Figure S.9a). As can be seen from Figure S.8a, the majority of the nCounter cartridges contained either four samples each from the three 4-hour conditions, or six samples each from the two 48-hour conditions; a distinct minority of cartridges contained samples from all five conditions. There were 46 technical duplicates (Figure S.9b), all taken in 2013.


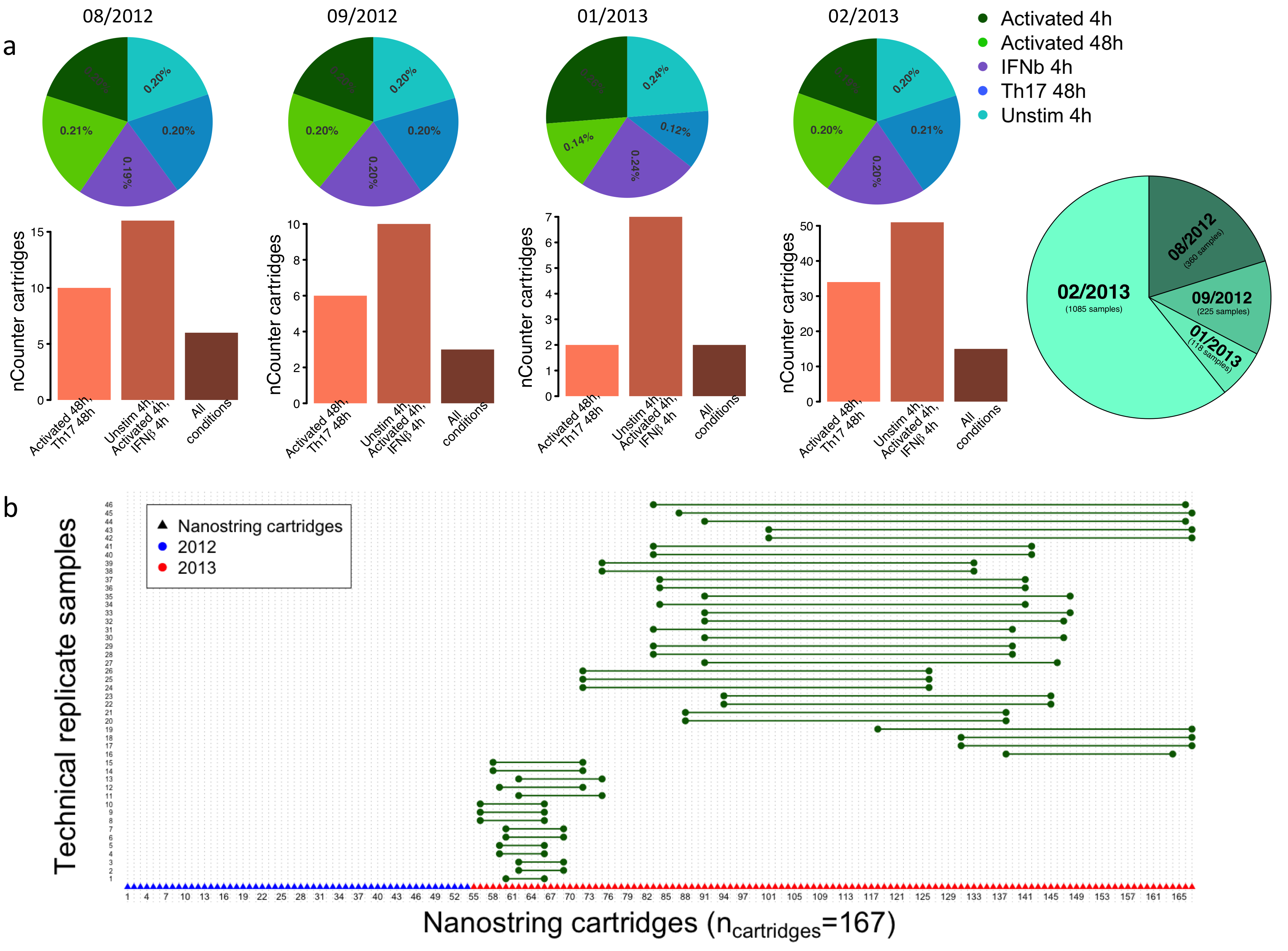


**Figure S9. Details of the T cell study. a)** Distribution of the different T cell sample conditions across different time points (pie plots). The majority of the nCounter cartridges contained either four samples each from the three 4-hour conditions, or six samples each from the two 48-hour conditions; a distinct minority of cartridges contained samples from all five conditions. **b)** Distribution of 46 technical replicate samples across cartridges. None of technical replicate samples spanned 2012 and 2013.

The authors selected 5 low variance control genes based on their microarray data, and carried out an nCounter normalization using these 5 (*ACTB, TBP, POLR2A, EIF5A and EEF1G*) as housekeeping genes and the 8 NEG and 6 POS transcripts, in one of the 84 options mentioned above. They did not mention how they used negative and positive controls, but they normalized data based on average of their housekeeping genes. After this they carried out a further variance stabilization transformation, which gave the data we’ll refer to below as Ye-normalized.


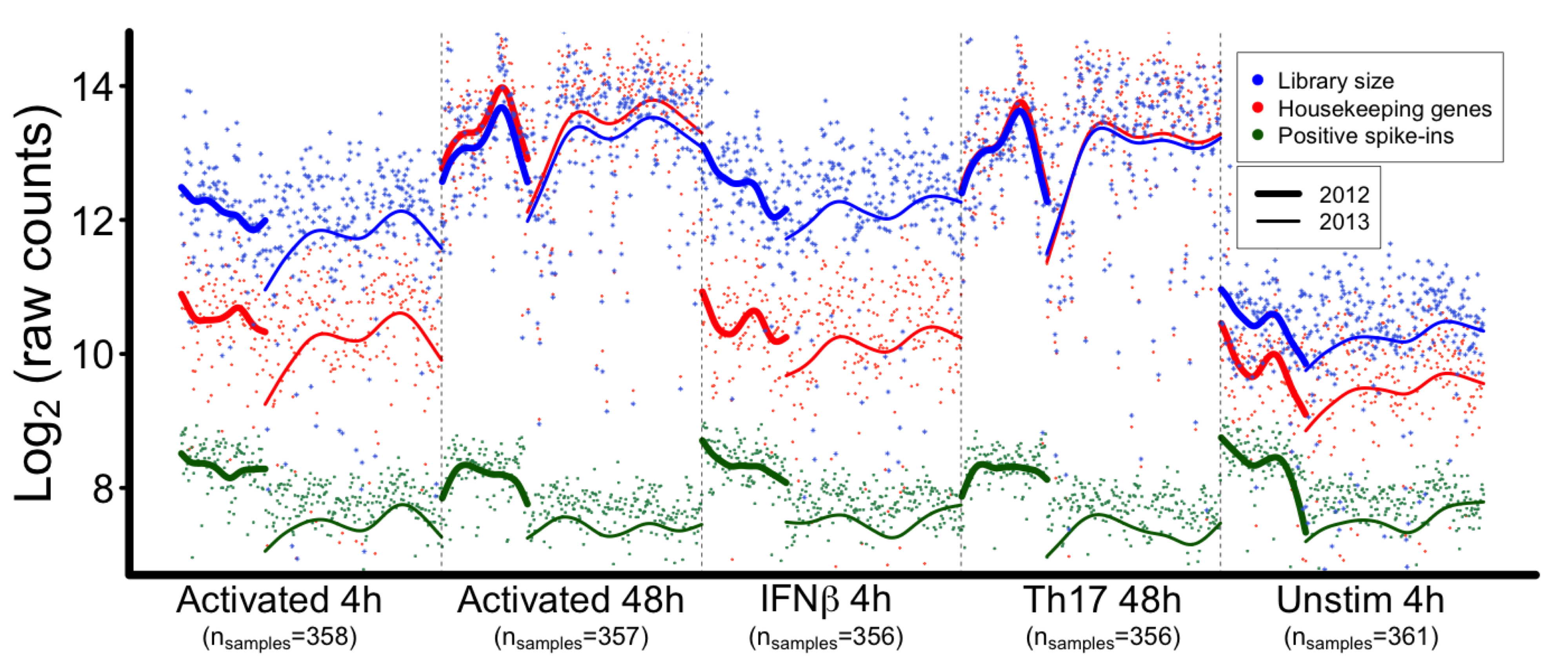


**Figure S10. Average plot of the T cell Nanostring data.** Samples in each condition were ordered according the time of running Nanostring cartridges, from 2012 to 2013. Samples with 48 hour conditions have higher library sizes compared to those from the other conditions. The positive spike-in controls are affected by time (2012 and 2013). The averages of the negative spike-in controls were quite stable across all cartridges (data not shown).

The RLE plots of the unnormalized Nanostring data (Figure S.11a) indicate a large amount of unwanted variation within and between the T cell conditions. The Ye- normalization led to a very poor RLE plot, with the two 48-hour and the three 4-hour conditions show striking shifts. RUV-III normalization using a set of 9 negative control genes largely removes this unwanted variation, and RLE plot of RUV-III normalized data is very similar to that of the microarray data using the Nanostring list of 236 genes.


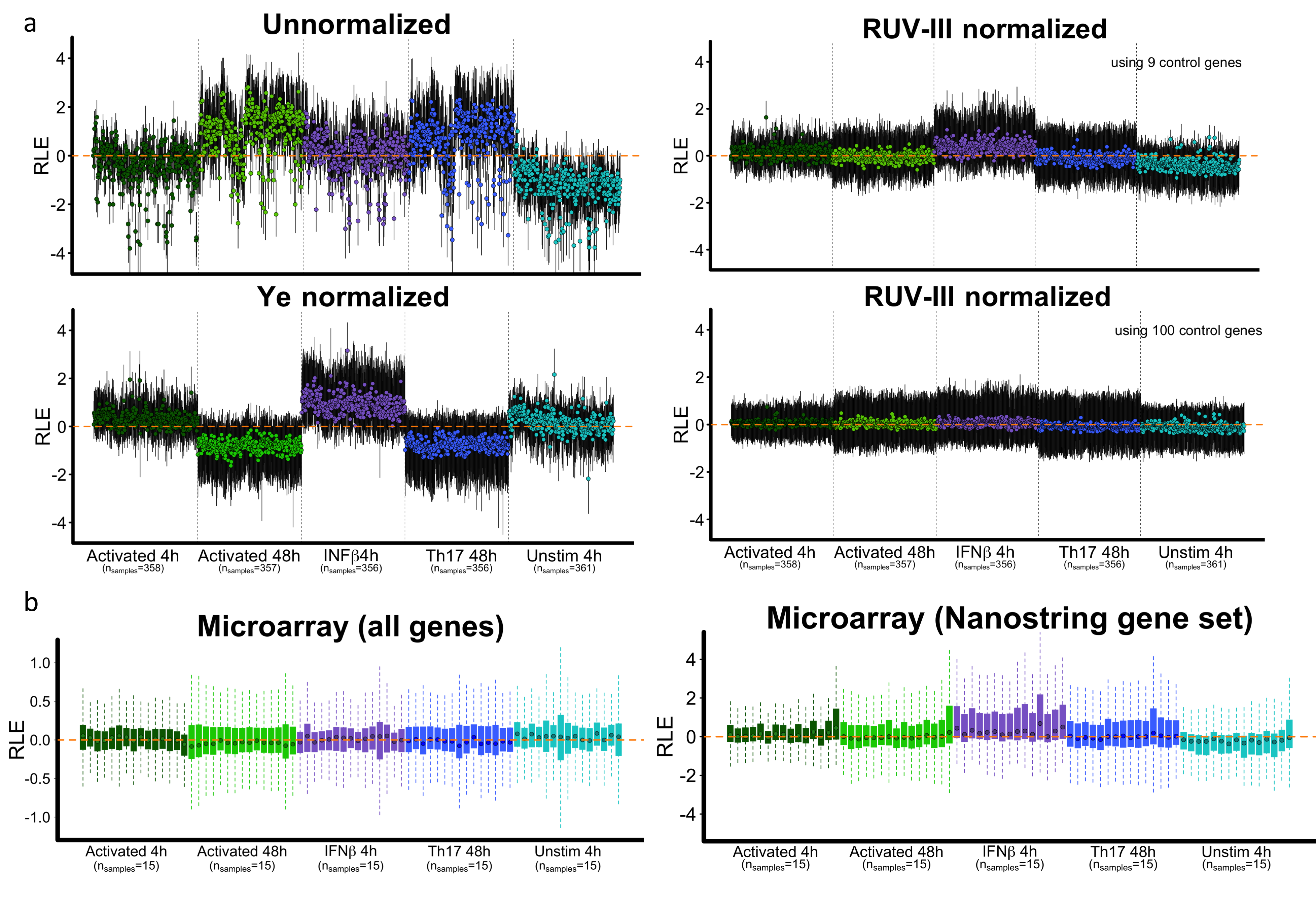


**Figure S11. RLE plots. a)** RLE plots of unnormalized data; Ye-normalized data and RUV-III normalized data using two different sets of negative control genes. The RLE plot of RUV-III normalized data using 9 negative control genes is very similar to that of the Ye *et al* microarray data with the Nanostring gene list. **b)** RLE plots of the Ye *et al* microarray data using all genes (left) and the Nanostring gene list (right).

Examining the expression patterns of housekeeping genes after using them for normalization is an important part of assessing the performance of normalization. Ideally we should see constant expression of these genes across experimental condition after normalization. However, the expression patterns of housekeeping genes in the Ye-normalized data are far from constant across the T cell conditions and they exhibit very different behaviour from that seen in the microarray data (Figure S.12).


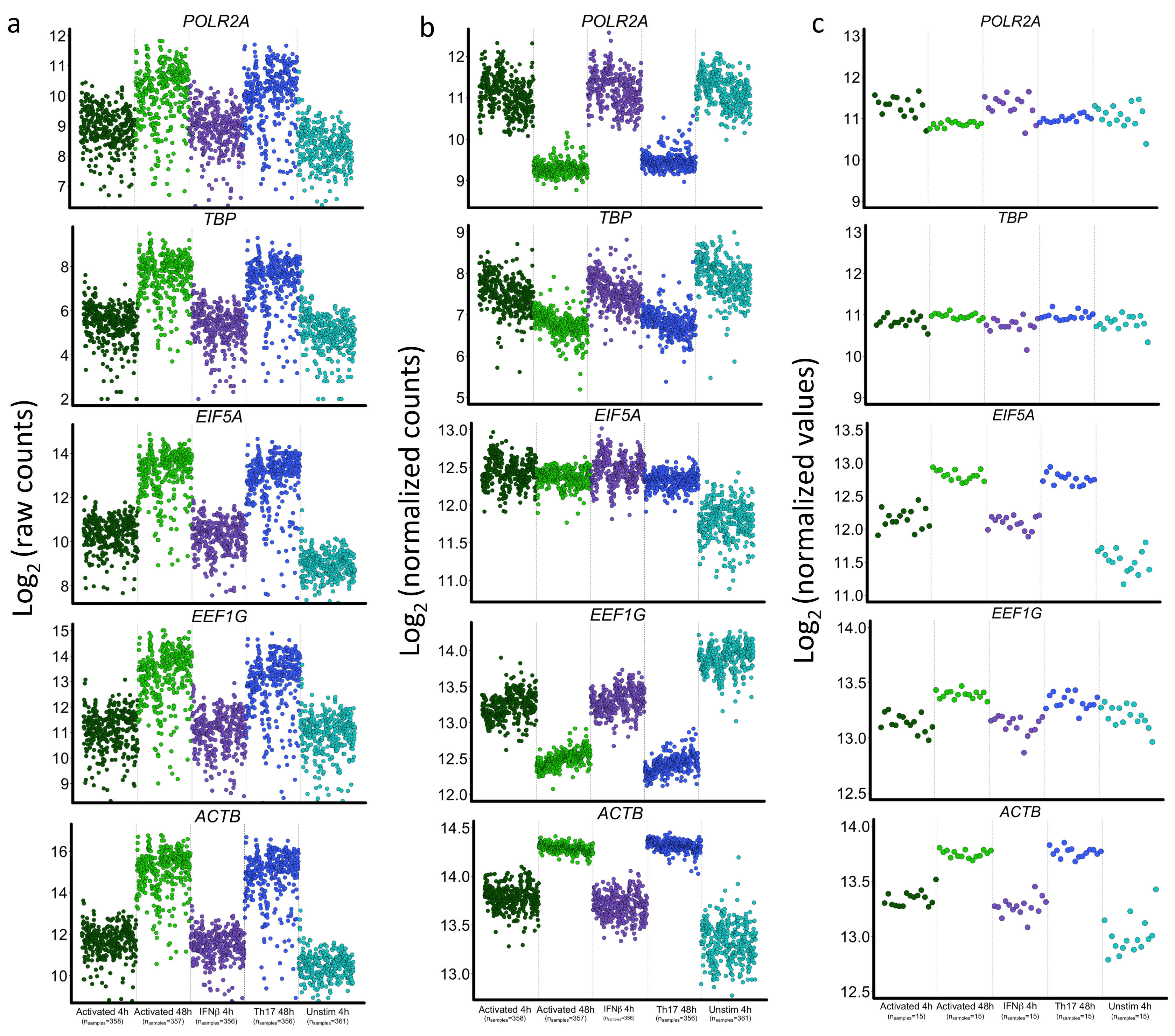


**Figure S12. Expression patterns of the Ye *et al* housekeeping genes. a)** Dot plots these genes in unnormalized Nanostring data. **b)** Dot plots of these genes in Ye *et al* -normalized data**. c)** Dot plots of these genes in the Ye *et al* microarray data.

To evaluate the RUV-III normalization and compare it to the Ye *et al* normalization, we carried out differential expression analyses between pairs of T cell conditions. We then examined the concordance between the log fold-changes obtained using the Ye*-*normalized and the RUV-III normalized data and the corresponding results from the microarray data (Figure S.13). Concordance here is taken to be similarity of estimated log fold changes between different conditions, including agreement in the direction of change. We repeated the same analysis for RUV-III with using 100 negative control genes (Figure S.14). We use the authors’ normalized microarray data directly from GEO.


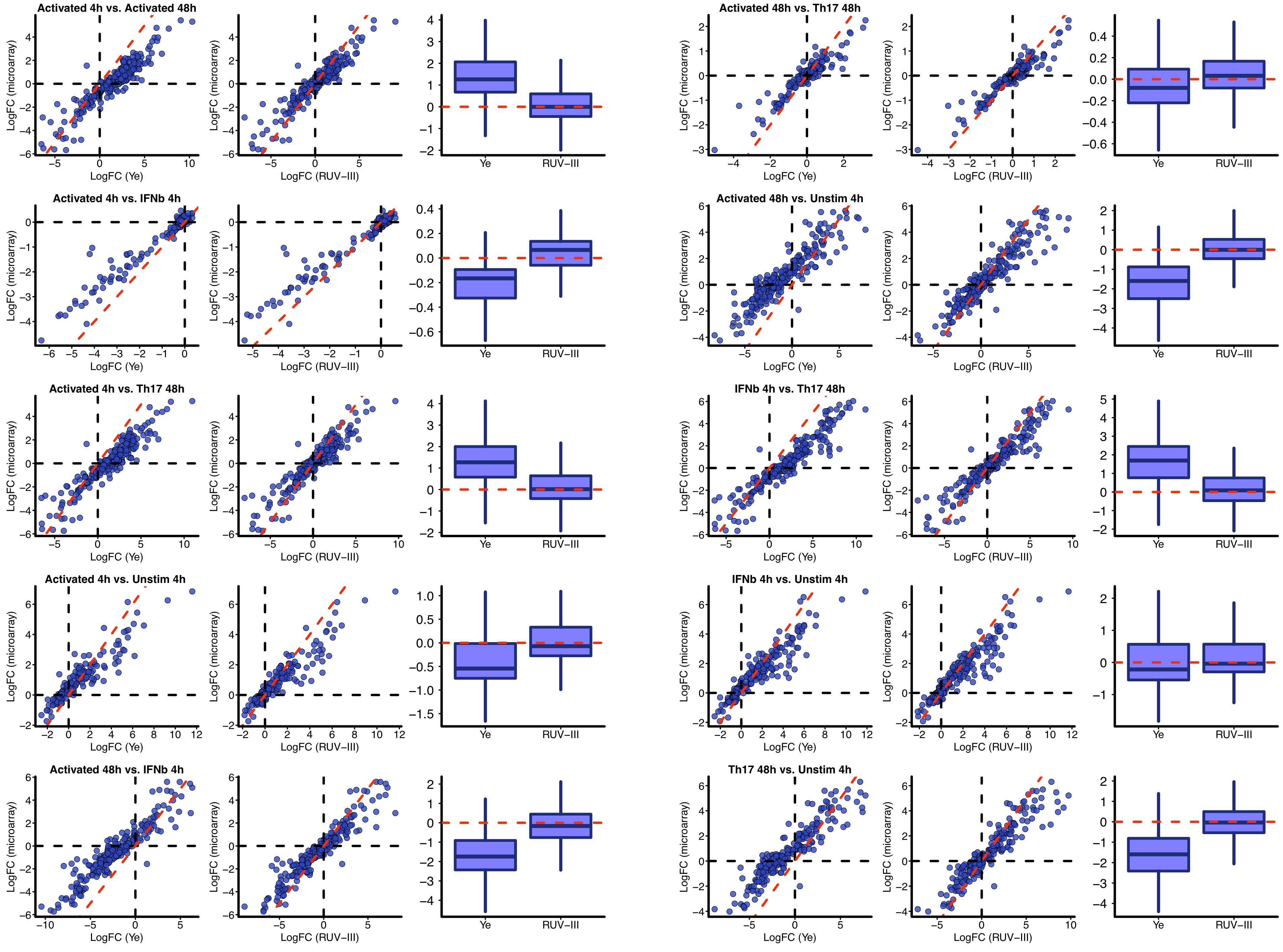


**Figure S13. Differential expression analysis between all pairs of T cell condition using Ye-normalized and RUV-III normalized data, comparing the estimated log fold changes with the corresponding results from the microarray data. Beside each pair of scatter plots we present box plots of the sets of differences:** log fold change (Ye) – log fold change (microarray) and log fold change (RUV-III) – log fold change (microarray). Here we used a set of 9 negative control genes for RUV-III.


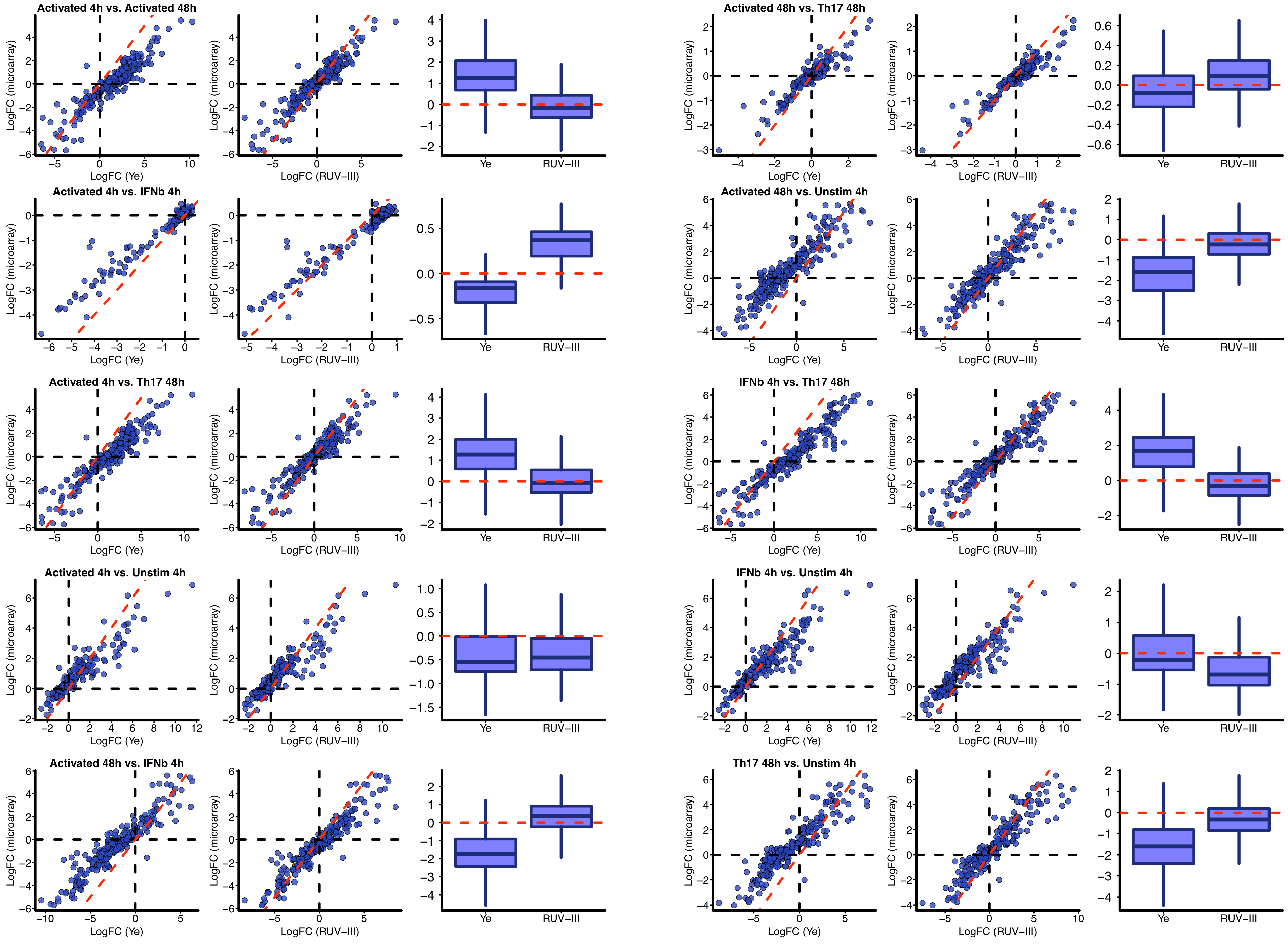


**Figure S14. Same a Figure S13.** Here we used a set of 100 negative control genes for RUV-III.

Our differential expression analysis showed that the expression patterns of a number of genes in Ye-normalized data were not concordant with that observed in RUV-III normalized and the microarray data. Figure S15 gives examples.


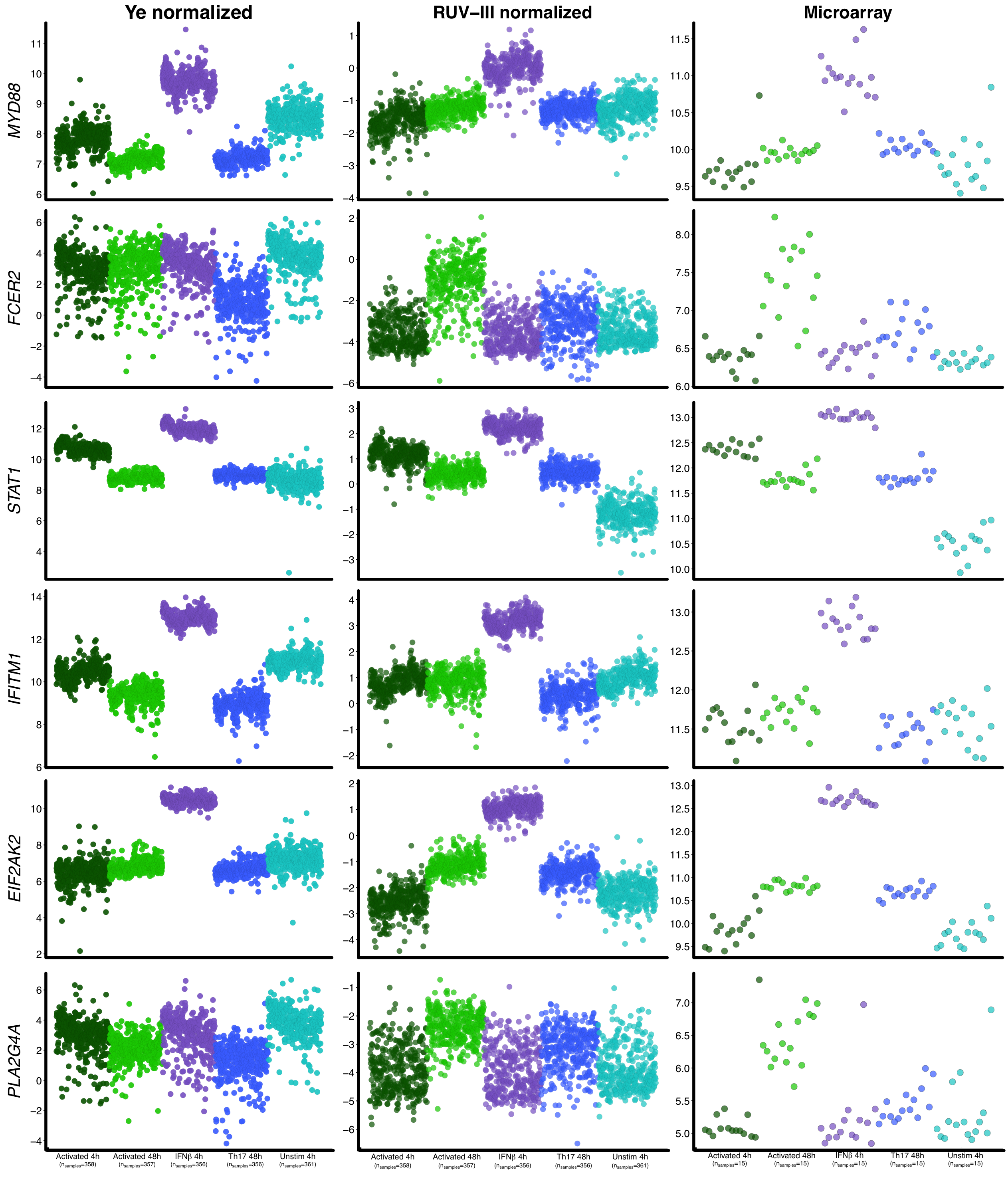


**Figure S15. Expression patterns of several genes across Ye-normalized and RUV-III normalized Nanostring data and the Ye *et al* microarray data.** These patterns are not consistent between the Ye-normalized data and RUV-III normalized data, whereas the latter is more similar to the Ye *et al* microarray data.

We would like to reiterate that our assessment is only of the normalization of Ye at al's  Nanostring data. As is explained in the supplementary material of Ye et al (2014), their population analyses were carried out with data adjusted for many covariates, including age, gender, BMI, weight, height, diastolic, systolic, seasonal, culture year, hybridization time, nCounter position, visit year, FCGR2A rs1801274 genotype status, and 7-9 principal components, with different numbers of components for different conditions.  Similar considerations applied to their eQTL mapping.

## Dendritic cell study

The goal of the Lee *et al* study [4] was to determine the effects of genetic variation on the induction of pathogen-responsive genes in human dendritic cells. It has a lot in common with the T cell study above and a broadly similar approach, but here it is based on the high-throughput isolation and activation of primary CD4^+^CD16^lo^ cells from peripheral blood to get monocyte-derived dendritic cells (MoDCs).

They began with microarray gene expression profiling of a limited number of subjects, and continued by profiling a reduced set of genes on a much larger group of subjects, using the Nanostring platform. The genome-wide microarray profiling was on 30 healthy donors, whose treated cells were profiled in three conditions: unstimulated (UNS) 0h, stimulated by *E. coli* lipopolysaccharide (LPS) 5h, and stimulated by influenza virus (FLU) 10h, while the larger study involved five conditions: UNS 0h, LPS 5h, FLU 10h, plus LPS 2.5h and stimulated by β-interferon (IFNβ) 6.5h. Lee *et al.* [4] do not discuss their LPS 2.5h data, and so we omit mention of these samples, although their data is available in GEO.

The Nanostring data set obtained from GEO for this study consisted of 2,441 nCounter assays obtained by profiling 676 healthy individuals across the five conditions for 414 genes (including different sets of control genes and 15 housekeeping genes) run over the years 2010-2012, with 258 technical replicates (285 extra assays of 2441), see Figure S15. The assays contained 16 housekeeping genes, these being made up of genes selected for their low variance in expression in the microarray data, genes with low variance in response to LPS or FLU stimulations, genes known not to be expressed in MoDCs, and three-sex specific genes (gender genes). It would seem that the Nanostring data here was normalized in the same way as was the data in the T cell study, but with the larger set of control genes as housekeeping genes, although the description in the paper makes no specific mention of the spiked-in NEG transcripts.

From the perspective of unwanted variation, several aspects of this study are noteworthy. First, the sample collection was spread over three years. Further, 848 of the original 2,441 assays were removed. We did our best to assemble the same Nanostring data set as that analyzed in the paper. We excluded all 308 samples from the LPS 2.5 h condition, as Lee et al did not mention these in their paper. We then removed 176 samples that had a technical duplicate, taking care to retain the one with the better set of expression measurements. In doing so, we considered library size and distribution of gene expression of each duplicate pair. We also removed several samples with incorrect annotation. Finally, we had to exclude 364 samples that Lee et al did not use for their analysis. We normalized 1769 (1593 unique assays and 176 extra assays) assays using RUV-III. Despite the presence of a large number of duplicates, this dataset proved extremely challenging to normalize. Especially problematic was the unwanted variation in the UNS condition.

In the course of trying to understand the unwanted variation in these data, we saw systematic differences in gene expression under the *same* condition (UNS 0h, LPS 5h etc) from treated blood samples from the *same* subject that were *used differently* in the study. Thus we created sample labels L, LVI, U etc, see Figure S.16a, according to whether an individual’s blood was assayed under the condition LPS 5h only (L), all three of LPS 5h, FLU 10h and INFβ (LVI), or UNS only (U), etc. We call these *usage types*. While we cannot explain how these striking expression differences came about, the evidence for them is compelling, see Figures S20 and S21.

As explained in the main text, we addressed this problem by defining 10 pairs of pseudo-replicates. These were samples spanning the U and ULVI samples types matched on gender and level (high and low) of four genes (*GSTT1, ZFP57, GP1BA* and *ERAP2*) with clear bimodal distributions, most likely major eQTL genes.


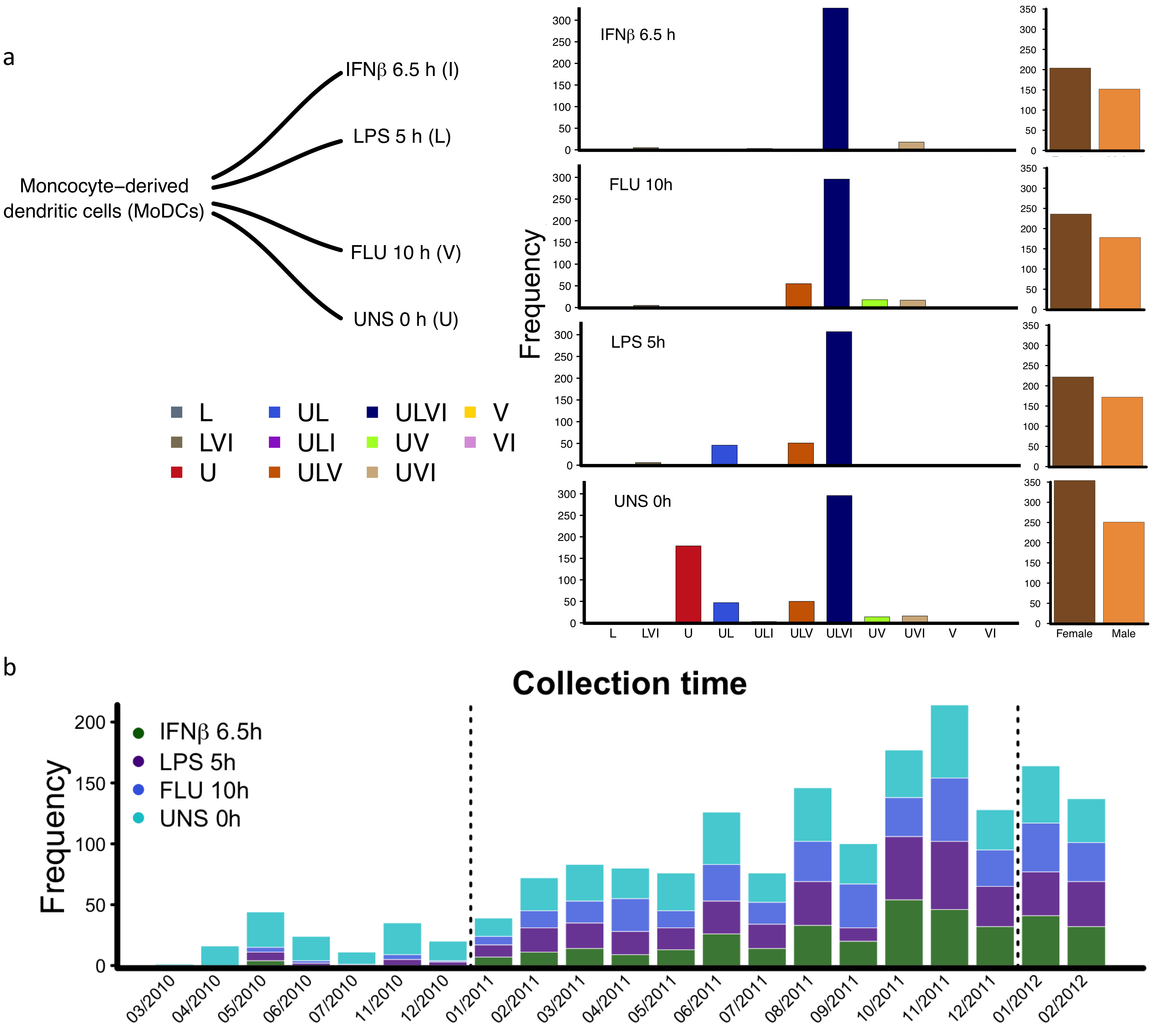


**Figure S16. Some details concerning the dendritic cell study samples. a)** Frequency of usage types for each condition. **b)** Frequency of samples collected across time, by condition.


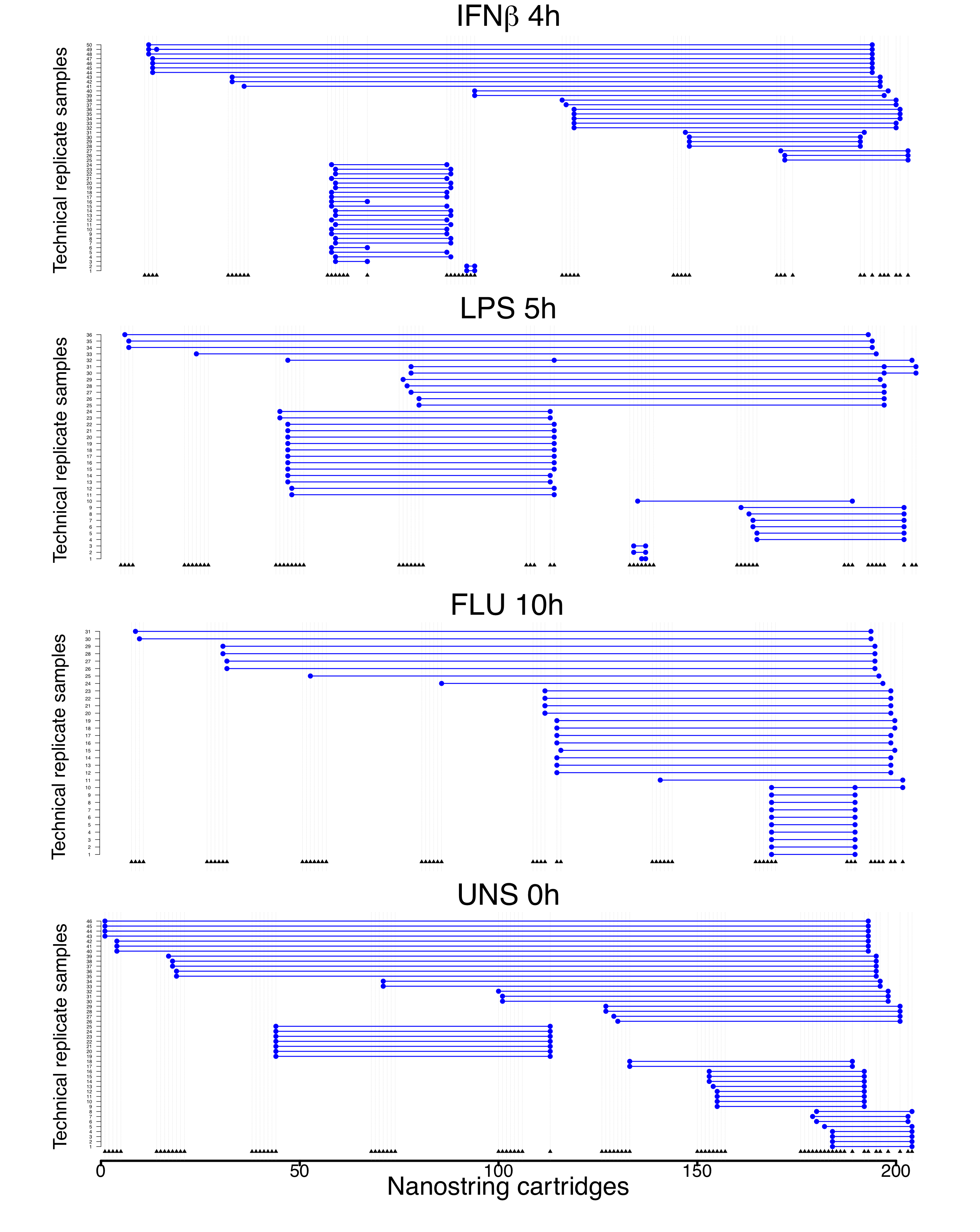


**Figure S17. Distribution of technical replicate samples across Nanostring cartridges within each condition.** Each triangle at the bottom of the figure represents a cartridge.


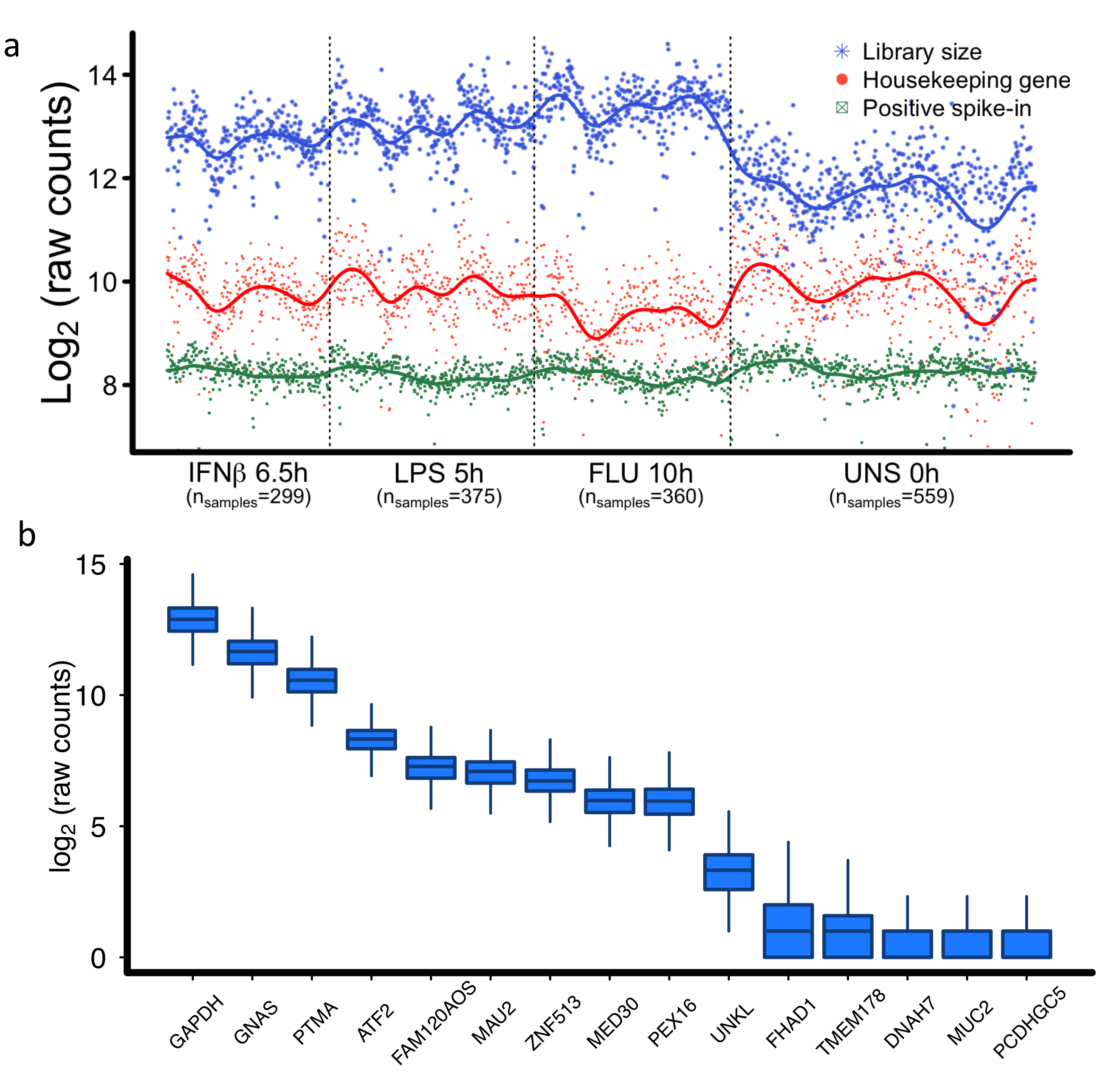


**Figure S18. Average plot and expression levels of housekeeping genes in the Lee *et al* Nanostring data. a)** Samples for each condition were ordered according the time of running the cartridges. The library size is slowly increasing from the INFß to the FLU samples, and drops dramatically for the UNS samples, whereas the expression levels of the housekeeping genes are relatively constant across apart from a noticeable decline in the FLU samples. **b)** Expression levels of the Lee *et al* housekeeping genes in the raw Nanostring data. Six genes show little or no expression.


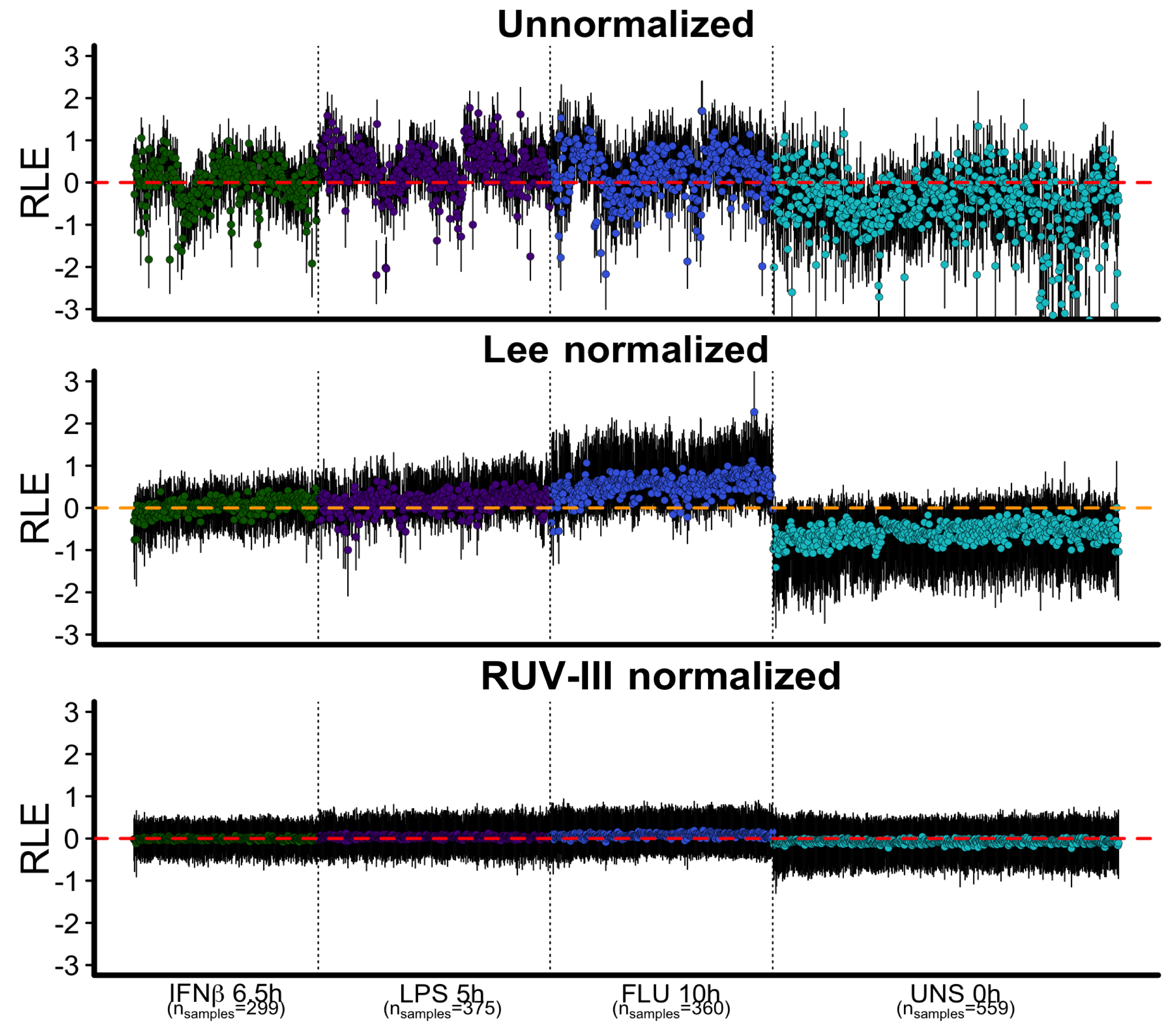


**Figure 19S. RLE for unnormalized, Lee-normalized and RUV-III normalized data.** Interquartile ranges and medians of RLE boxplot are presented here. Samples are grouped according to condition and are ordered within condition according to the time of running cartridges.


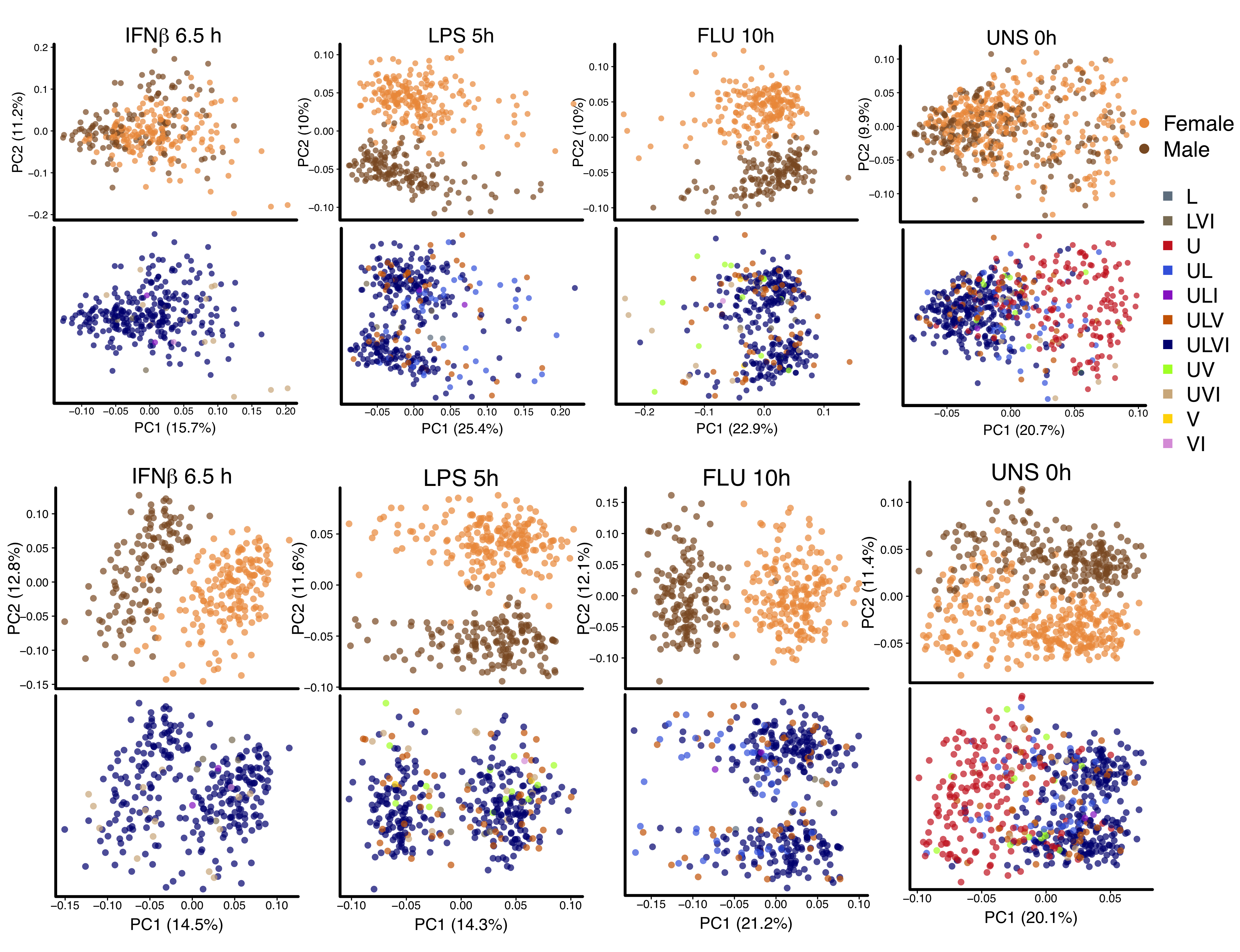


**Figure S20. Scatter plots of the first two principal components for the Lee-normalized data and RUV-III normalized data without using any pseudo replicate samples.**


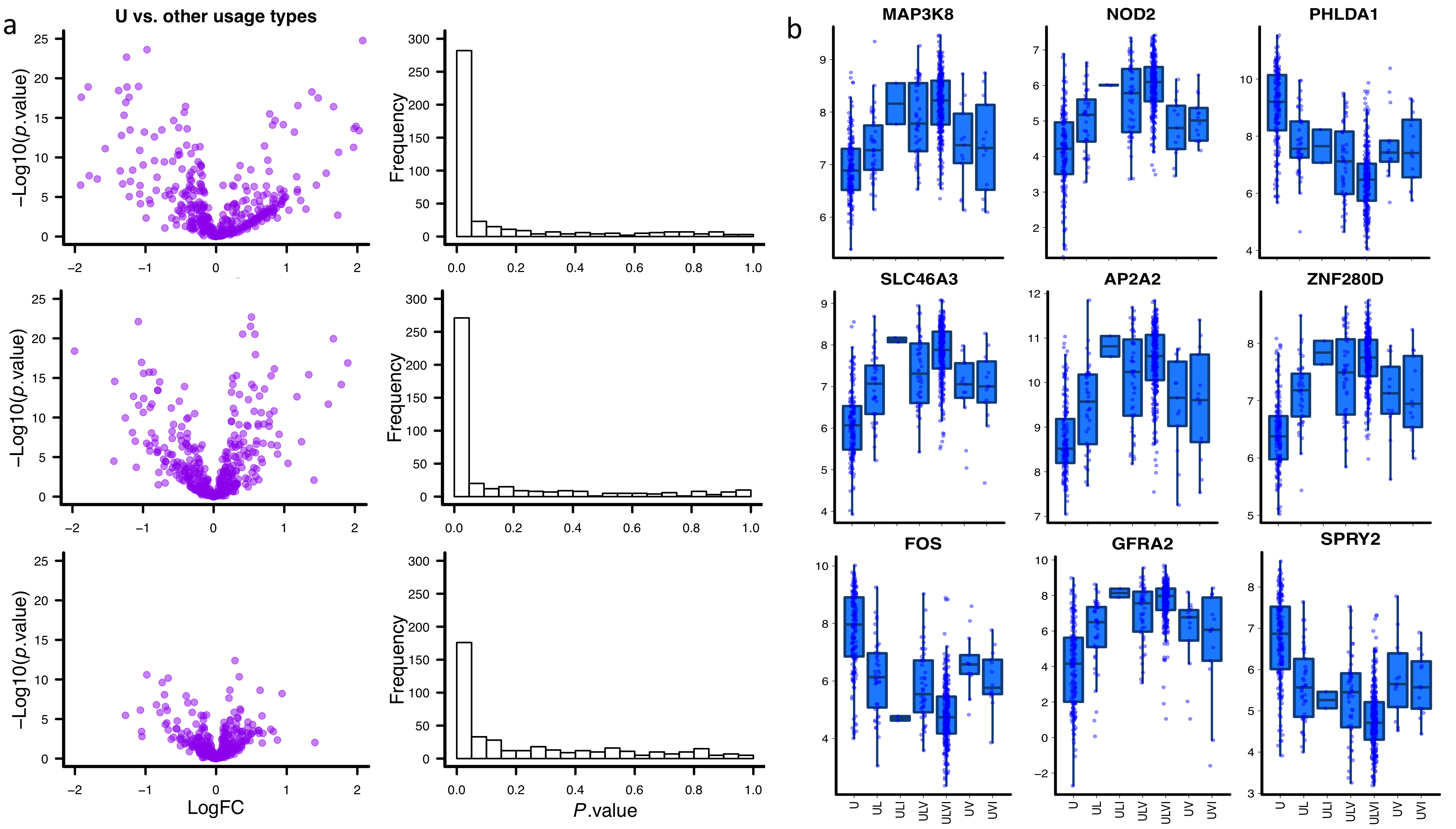


**Figure S21. Technical variation in unstimulated conditions. a)** Volcano plots and *p*-value histograms following differential expression analysis between U and all other usage types of unstimulated samples for Lee-normalized data (top), RUV-III normalized data (middle) and RUV-III with 10 pairs of pseudo technical replicates (bottom). **b)** Expression patterns of selected genes differentially expressed between U and all other usage types of unstimulated samples in Lee-normalized Nanostring data.

# Workflow

We present (Figure S.22) a step-by-step workflow of RUV-III normalization for Nanostring data sets with technical replicate samples. The input gene expression matrix (samples are in rows and genes are in columns) for RUV-III normalization should be log transformed raw Nanostring counts. An appropriate set of negative control genes (usually beginning with all genes) and number k should be selected. Three levels of evidence are used to assess whether the normalization has succeeded. These include: weak evidence (e.g. see a good RLE or PCA plot); intermediate evidence (e.g. good technical replicate agreement plots); and strong evidence (typically recapitulating known biology). Varying the number and nature of the negative control genes and the number k, and, after a deeper examination of the data, perhaps creating pseudo-replicates, may be required if an initial RUV-III normalization do not seem to be satisfactory.


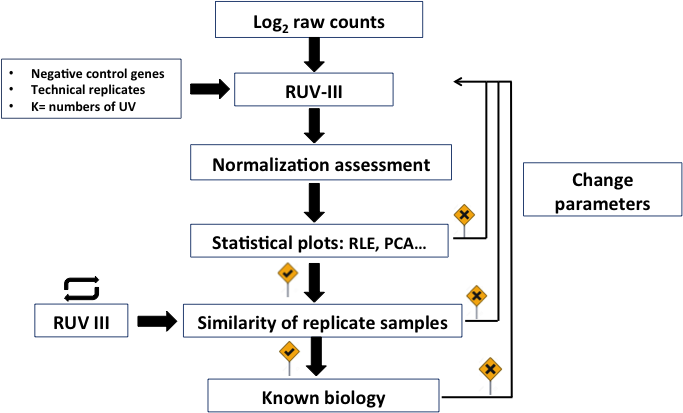


**Figure S22. Step by step workflow for applying RUV-III to Nanostring data with technical replicate samples.**

# Supplementary method

## RUV-III model

Suppose we have data from $m$ Nanostring assays on *m’< m* biologically distinct samples. The condition *m’ < m* implies that at least some of the samples were assayed more than once, i.e. some of the assays are technical replicates. Let $M$ be the $m\times m\mathbf{'}$ *mapping matrix* that maps assays to samples. More specifically, the $(i, j)$-th entry of $M$ is 1 if the $i$-th assay is an assay of sample $j$, and 0 otherwise.

Suppose that the assays have $n$ probes. Most of the probes correspond to real genes, but some may correspond to the ERCC spike-ins, exogenous (e.g. bacterial or viral sequences), while some may be random sequences that do not correspond to any organism. The ERCC spike-in probes are sometimes referred to as "positive controls" since the ERCC spike-ins are known to be present and the random-sequence probes are sometimes referred to as "negative controls" because they should not bind to anything in the mRNA pool. However, this terminology conflicts with our own. We use the term *negative controls* to refer to probes whose expression values *should not* be influenced by the biological factor(s) of interest; this definition will be made more precise below. Conversely, we use the term *positive controls* to refer to probes whose expression values *should* be influenced by the biological factor(s) of interest. Note that under our terminology, both ERCC spike-ins and random-sequence probes may be considered negative controls, since the measured expression values of each should remain constant across assays and thus be uninfluenced by the biological factor of interest.

Let $Y$ be the $m\times n$ matrix of observed expression values (log counts). We model $Y$ as

$Y_{m\times n}=X_{m\times p}\beta_{p\times n}+W_{m\times k}\alpha_{k\times n}+\epsilon_{m\times n}$ (1)

where $X\beta$ is the biological variation of interest, $W\alpha$ is unwanted variation, $\epsilon$ is random error, and we assume that $p+k<m$ and $k<m-m'$.

More specifically, $X$ corresponds to the biological factor(s) of interest (e.g. disease state, tumor sub-type, etc.) and not technical factors such as batch. *X* may or may not be observed. For example, in a differential expression analysis where the goal is to determine which genes are differentially expressed between different disease states, $X$ would represent disease state and would be known; the goal of the analysis would be to estimate $\beta$. Alternatively, in a clustering analysis where the goal^[[2]](#footnote-2)^ is to discover new tumor sub-types, $X$ would represent the (unknown) subtypes and would be unobserved. In what follows, our focus will be on the case in which $X$ is unobserved, as it is here that we make essential use of technical replicates. The unwanted factors $W$ are assumed to be unobserved as well.

Note that if assays $i$ and $i'$ are technical replicates of sample $j$, then rows $i$ and $i'$ of $X$ should be identical, since the biological sample in both assays is the same. We may therefore write

$$X=M\mathbf{X}$$

where we have implicitly defined $\mathbf{X}_{m\mathbf{'}\times p}$ as the biological factor of interest in terms of samples rather than arrays.

Finally, we assume that a number $n_{c}<n$ of the probes are *negative control* probes. We indicate these probes by a subscript $c$. More specifically, we let $Y_{c}$ be the $m\times n_{c}$ submatrix of $Y$ whose columns contain only the negative control probes. Similarly $\beta_{c}$ is a $p\times n_{c}$ submatrix of $\beta$, $\alpha_{c}$ is a $k\times n_{c}$ submatrix of $\alpha$, and $\epsilon_{c}$ is a $m\times n_{c}$ submatrix of $\epsilon$. The *negative control assumption* is that

$$\beta_{c}=0.$$

This is the assumption that the negative controls are uninfluenced by the biological factor(s) of interest. We also assume that $k\leq n_{c}$ and that $\alpha_{c}$ is full rank (see comments below).

# Method

Our goal is to estimate the $W\alpha$ term in (1) and subtract it off from $Y$, leaving us with an adjusted matrix $\overset{̂}{Y}$ that may be used for downstream analysis.

Let

$$R_{M}=I-M\left( M'M \right)^{-1}M'$$

be the residual operator of M. Then

$$\begin{matrix} R_{M}Y= & R_{M}(X\beta+W\alpha+\epsilon) \\ = & R_{M}M\mathbf{X}\beta+R_{M}W\alpha+R_{M}\epsilon\\ = & R_{M}W\alpha+R_{M}\epsilon\end{matrix}$$

which suggests that we may estimate $\alpha$ by some form of factor analysis on $R_{M}Y$, so long as $R_{M}W$ is full rank (see comments below). We therefore let $\overset{̂}{\alpha}$ be the first $k$ right singular vectors of $R_{M}Y$. Note that in practice $k$ is unknown and must be selected by the researcher (see comments below).

Having estimated $\alpha$ we now estimate $W$. We let

$$\overset{̂}{W}=Y_{c}{\overset{̂}{\alpha}}_{c}'\left( {\overset{̂}{\alpha}}_{c}{\overset{̂}{\alpha}}_{c}' \right)^{-1}.$$

Note that

$$\begin{matrix} \overset{̂}{W}= & Y_{c}{\overset{̂}{\alpha}}_{c}'\left( {\overset{̂}{\alpha}}_{c}{\overset{̂}{\alpha}}_{c}' \right)^{-1} \\ = & (X\beta_{c}+W\alpha_{c}+\epsilon_{c}){\overset{̂}{\alpha}}_{c}'\left( {\overset{̂}{\alpha}}_{c}{\overset{̂}{\alpha}}_{c}' \right)^{-1} (2) \\ = & \left( W\alpha_{c}+\epsilon_{c} \right){\overset{̂}{\alpha}}_{c}^{'\left( {\overset{̂}{\alpha}}_{c}{\overset{̂}{\alpha}}_{c}' \right)^{-1}} (3) \\ = & W\alpha_{c}{\overset{̂}{\alpha}}_{c}'\left( {\overset{̂}{\alpha}}_{c}\overset{̂}{\alpha}'_{c} \right)^{-1}+\epsilon_{c}{\overset{̂}{\alpha}}_{c}'\left( {\overset{̂}{\alpha}}_{c}{\overset{̂}{\alpha}}_{c}' \right)^{-1} \\ \approx& W+\epsilon_{c}{\overset{̂}{\alpha}}_{c}'\left( {\overset{̂}{\alpha}}_{c}{\overset{̂}{\alpha}}_{c}' \right)^{-1} \\ \approx& W. \end{matrix}$$

Finally, having estimated $W$ and $\alpha$, we let our adjusted matrix $\overset{̂}{Y}$ be

$$\overset{̂}{Y}=Y-\overset{̂}{W}\overset{̂}{\alpha}.$$

# Comments

As noted above, we require $\alpha_{c}$ to be full rank; in practice this means that the negative controls must be affected by the unwanted factors. For example, if temperature is an unwanted factor, then the negative controls should be affected by temperature. Otherwise the temperature factor (a column of $W$) would not be identifiable.

Similarly, we require $R_{M}W$ to be full rank, since otherwise some rows (or linear combinations of rows) of $\alpha$ would not be identifiable. In practice, this means that the technical replicates must "capture" the unwanted variation, in the sense that the unwanted factors must vary across the replicates. For example, if temperature is an unwanted factor, then there must be at least one set of replicates where temperature varies between the replicates. Otherwise, the temperature "signature" (a row of $\alpha$) could not be recovered.

Also as noted above, the number $k$ of unwanted factors is generally unknown and must be estimated. Our general approach, advocated by [5], is to repeat the analysis with multiple values of $k$, and then assess the quality of each analysis using various quality metrics and prior biological knowledge, as explained above. For example, in a clustering analysis one could check how well the samples cluster by known biology, or in a differential expression analysis, one could check that positive control probes are highly ranked or that the estimated log-fold differential expression correlates well with the log-fold differential expression observed in independent studies.

Another point to be made is that the negative control assumption $\beta_{c}=0$ is somewhat stronger than necessary. In moving from equation (2) to (3) we drop the $X\beta_{c}{\overset{̂}{\alpha}}_{c}'\left( {\overset{̂}{\alpha}}_{c}{\overset{̂}{\alpha}}_{c}' \right)^{-1}$ term because it is equal to 0. This is certainly true if $\beta_{c}=0$, but is also possible if $\beta_{c}\neq0$ as long as $\beta_{c}{\overset{̂}{\alpha}}_{c}'=0$. We interpret this to mean that RUV-III is reasonably robust to violations of the negative control assumption (for further discussion, see [6]). It is this observation that motivates us to use all genes as negative controls in some of the examples of this paper.

Finally, we note that RUV-III is essentially a variant of the replicate method presented in [7]. The main difference between the methods is the way in which replicate assays are used to construct a matrix of assay differences that contain only unwanted variation. In the method presented in [7], all pair-wise differences between technical replicates are considered. This effectively gives higher weight to replicates that are all assays of one sample than it does to an equal number of replicates that are assays of multiple samples. For example, one set of quadruplicates would receive higher weight than two sets of duplicates, because in the case of the quadruplicates, each of the assays would show up in three pair-wise differences, whereas in the case of duplicates each of the assays would show up in just one pair-wise difference. In RUV-III, however, instead of taking pair-wise differences, the mean of each set of replicates is subtracted off, leaving us with a matrix of "assay residuals" instead of "assay differences." This results in all replicates being weighted equally. RUV-III is also very similar formally to RUV-4 [6]. RUV-III is essentially equivalent to the first three steps of RUV-4, with the exception that we substitute the mapping matrix $M$ for $X$. It is because of this similarity with RUV-4 that we give RUV-III its name. (The use of "III" instead of "3" is a subtle reminder that unlike RUV-2 [5] or RUV-4, RUV-III is not intended primarily for differential expression analyses. It also distinguishes our use from the RUV3 in [8])

**References**

1. Waggott, D., et al., *NanoStringNorm: an extensible R package for the pre-processing of NanoString mRNA and miRNA data.* Bioinformatics, 2012. **28**(11): p. 1546-8.

2. Peloquin, J.M., et al., *Characterization of candidate genes in inflammatory bowel disease-associated risk loci.* JCI Insight, 2016. **1**(13): p. e87899.

3. Ye, C.J., et al., *Intersection of population variation and autoimmunity genetics in human T cell activation.* Science, 2014. **345**(6202): p. 1254665.

4. Lee, M.N., et al., *Common genetic variants modulate pathogen-sensing responses in human dendritic cells.* Science, 2014. **343**(6175): p. 1246980.

5. Gagnon-Bartsch, J.A. and T.P. Speed, *Using control genes to correct for unwanted variation in microarray data.* Biostatistics, 2012. **13**(3): p. 539-52.

6. Johann A Gagnon-Bartsch, L.J., and Terence P Speed, *Removing unwanted variation from high dimensional data with negative controls.* 2013(Department of Statistics, University of California at Berkeley, Technical Report #820).

7. Jacob, L., J.A. Gagnon-Bartsch, and T.P. Speed, *Correcting gene expression data when neither the unwanted variation nor the factor of interest are observed.* Biostatistics, 2016. **17**(1): p. 16-28.

8. Gerad, D., Stephens, M., *Unifying and generalizing methods for removing unwanted variation based on negative Controls.* eprint arXiv:1705.08393.

|  |
| --- |

1. We identify samples processed with a given reagent lot as a batch. For clarity we adopted the numbering 2, 3 and 4 for the reagent lots (which we call batches) used with the data downloaded from GEO rather than the 1, 2 and 3 used in the paper. It seems likely that these correspond 2-1, 3-2 and 4-3, but we are not certain. [↑](#footnote-ref-1)
2. In this case existing RUV options not needing technical replicates [5, 6, 7]. [↑](#footnote-ref-2)
